# Genome-wide analyses reveals an association between invasive urothelial carcinoma in the Shetland sheepdog and *NIPAL1*

**DOI:** 10.1038/s41698-024-00591-0

**Published:** 2024-05-22

**Authors:** Heidi G. Parker, Alexander C. Harris, Jocelyn Plassais, Deepika Dhawan, Erika M. Kim, Deborah W. Knapp, Elaine A. Ostrander

**Affiliations:** 1 Cancer Genetics and Comparative Genomics Branch, National Human Genome Research Center, National Institutes of Health, Bethesda, MD USA; 2 Institut de Génétique et Développement de Rennes, CNRS-UMR6290, University of Rennes, 35000 Rennes, France; 3 Department of Veterinary Clinical Sciences, College of Veterinary Medicine, Purdue University, West Lafayette, IN USA; 4 Center for Biomedical Informatics & Information Technology, National Cancer Institute, National Institutes of Health, Frederick, MD USA; 5 Purdue University Center for Cancer Research, West Lafayette, IN USA

**Keywords:** Bladder cancer, Cancer genetics, Cancer genomics

## Abstract

Naturally occurring canine invasive urinary carcinoma (iUC) closely resembles human muscle invasive bladder cancer in terms of histopathology, metastases, response to therapy, and low survival rate. The heterogeneous nature of the disease has led to the association of large numbers of risk loci in humans, however most are of small effect. There exists a need for new and accurate animal models of invasive bladder cancer. In dogs, distinct breeds show markedly different rates of iUC, thus presenting an opportunity to identify additional risk factors and overcome the locus heterogeneity encountered in human mapping studies. In the association study presented here, inclusive of 100 Shetland sheepdogs and 58 dogs of other breeds, we identify a homozygous protein altering point mutation within the *NIPAL1* gene which increases risk by eight-fold (OR = 8.42, CI = 3.12–22.71), accounting for nearly 30% of iUC risk in the Shetland sheepdog. Inclusion of six additional loci accounts for most of the disease risk in the breed and explains nearly 75% of the phenotypes in this study. When combined with sequence data from tumors, we show that variation in the MAPK signaling pathway is an overarching cause of iUC susceptibility in dogs.

## Introduction

Bladder cancer is the sixth most common human cancer in the United States, accounting for over 80,000 new cases per year (http://globocan.iarc.fr)^1^. Ninety percent of bladder cancers are urothelial carcinomas (UC), and while the noninvasive form is most common, approximately 25–30% are muscle invasive (MIBC) at the time of diagnosis^2,3^. Distant metastases are present in 5% of all cases at diagnosis, a number that ultimately grows to 50% for muscle-invasive disease^2,4,5^. Despite improvements in treatment, such as immune-based therapy and new methods for early diagnosis, the five-year survival rate for MIBC patients is only 58%, and 5% if the disease has metastasized. The risk of developing bladder cancer in humans can be attributed to several factors including smoking^6,7^, pollutants^8,9^, and genetic risk variants^10,11^.

Domestic dogs develop cancer naturally as they age, just as humans do, are diagnosed as symptoms arise, and are treated with surgery, radiation, and/or chemotherapy. The increased risk of specific cancers observed in subsets of breeds indicates the presence of inherited mutations of high penetrance that are conducive to genome wide association studies (GWAS). The reasons for this are several-fold. Importantly, breed structure requires that any dog who is a registered member of a breed must be descended from registered members of the same breed, thus forming closed populations segregating limited numbers of mutations. Also, most breeds feature popular sires whose genomes are over-represented in the breed population, further ensuring that small numbers of variants are likely responsible for increased susceptibility to a particular cancer. Together, this means that canine cancer genetics represents a unique approach for dealing with the problem of locus heterogeneity that is so common in genetic studies of human cancer susceptibility. Finally, canine cancers are not induced. This ensures that the loci identified contribute to susceptibility and disease development rather than other aspects of the tumor life cycle. Indeed, while other approaches, such as those based on murine/PDX, knock-out mice, CRISPR generated mutation driven tumors, etc., exist for studies of tumor metastasis, growth rate, clonality, and treatment response, the canine system proves most useful in revealing the genetic underpinnings of disease susceptibility, especially for traits where multiple genes contribute differentially, such as cancer.

Invasive urinary carcinoma (iUC) is the most common cancer of the urinary bladder in dogs, accounting for 90% of bladder cancers, with 40,000 newly diagnosed cases a year^12,13^. Naturally occurring canine iUC closely mimics human high grade MIBC in terms of age of onset, pathology, cellular and molecular features, metastatic profiles, and treatment response^14^. Approximately 50% of affected dogs will experience metastases to distant organs^15^ and median survival times are typically < one year, although dogs receiving multiple sequential protocols may live beyond a year^16,17^. As with humans, there are multiple environmental factors that reportedly increase risk of canine iUC^18^. Approximately 80% of canine iUC tumors carry a somatic BRAF^V595E^ mutation, which has been shown to increase activity of the MAPK signaling pathway^19,20^. Similarly, more than one third of human MIBC tumors carry activating mutations in the MAPK pathway^21^. Tumor cells treated with therapeutics that target a specific somatic mutations can develop resistance through multiple mechanisms (reviewed in^22^). For example, in response to MAPK pathway inhibitors that target the BRAF^V600E^ mutation, tumor cells have been found to reactivate the MAPK pathway through mutations in the tyrosine kinase receptors or RAS genes or by duplicating and further mutating the BRAF gene^23,24^. Treatment options are improved when the pathway is treated as a whole.

Amongst purebred dogs, there is a subset of breeds at increased risk for developing iUC, including the Scottish terrier (OR = 21.1, 95% CI = 16.2–27.5), West Highland white terrier (OR = 5.8 95% CI = 4.2–8.1), and Shetland sheepdog (OR = 6.1, 95% CI = 4.8–7.7)^12^. Shetland sheepdogs (Shelties), the focus of this study, are a small herding breed and popular family pet. Multiple studies demonstrate their extraordinary disease risk^12,17,25^, with one survey reporting that of 3359 Shelties (https://ofa.org/about/health-surveys/?breed=SS), iUC comprised 12% of all Sheltie cancer diagnoses, which is six times the expected rate of 2% for all dogs^13^. Of note, dogs are more likely to get invasive disease rather than non-invasive disease and tumors are typically stage T2 or higher at diagnoses^25–27^. These facts, combined with the high level of veterinary scrutiny dogs receive, are additional attributes making domestic dog breeds an ideal system for iUC genetic mapping studies.

In this study we analyze one hundred Shelties and 55 dogs from closely related breeds and identify a single major risk locus associated with iUC, featuring *NIPA-like domain containing 1* (*NIPAL1)*, a strong candidate gene, and associated mutations. By segregating samples according to genotype, we identify six additional loci that modify individual disease risk when combined with a putative pathogenic protein altering mutation in *NIPAL1*. Merging the GWAS loci with tumor sequence identifies the mitogen activated protein kinase (MAPK) signaling pathway as a key component of canine iUC genetic susceptibility.

## Results

### Genome-wide association study

**Fig. 1:**
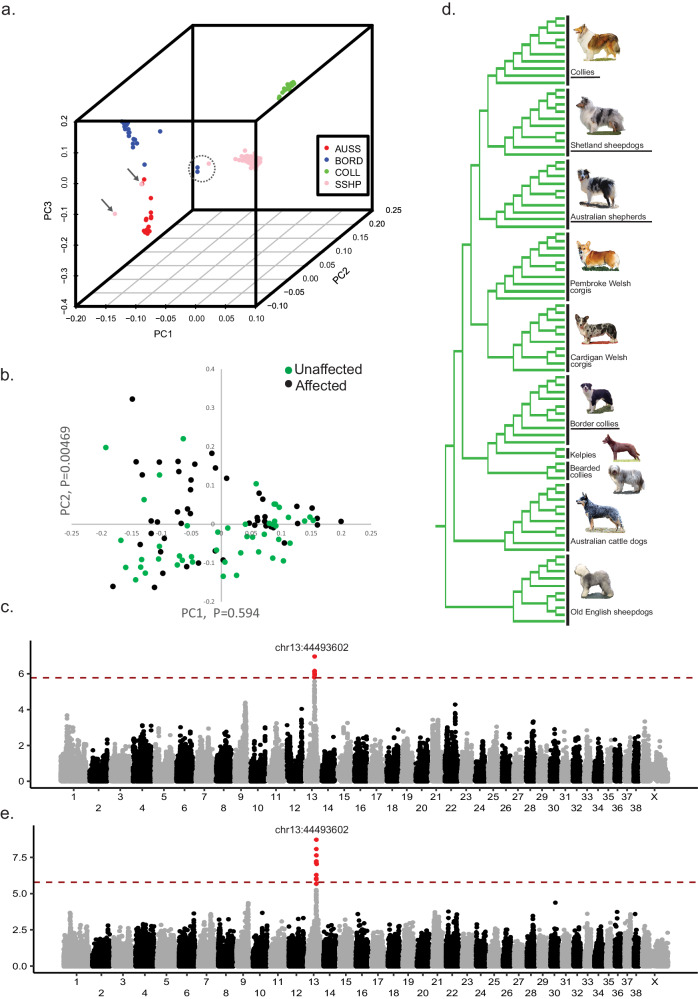
Sample choice and results from GWAS of iUC in Shelties and other herding breeds. **a** PC plot of four herding breeds: Sheltie, Collie, Border collie and Australian shepherd. Arrows indicate mis-assigned dogs that were removed from the study. Circle encloses three samples in between breeds determined atypical of their reported breed. For clarity, Supplementary Figure 1 shows two-dimensional plots of the same three PCs in all three combinations. **b** PC plot of only Shetland sheepdogs with iUC cases and controls highlighted. *P*-values for correlation with disease status are given along the axes. **c** Manhattan plot showing association values of 90,530 SNPs with iUC in 100 Shetland sheepdogs. **d** Breed tree showing the breeds that form the UK herding clade from (Parker et al. 2017) with breeds used in the GWAS analysis underlined. **e** GWAS of 156 herding dogs from the four related breeds. The x-axis comprises SNP positions from the beginning of chr1 through the end of the X chromosome. Shading of black and gray shows chromosome boundaries. The y-axis displays the -log of the *p*-value. The red line indicates the Bonferroni significance estimate, 1.7 × 10^−6^. Position of the top SNP at each significant locus is indicated above it. SNPs with suggestive *p*-value less than 1 × 10^−5^ are highlighted in red.

Affected Shelties were submitted to the study through the Purdue University Veterinary Hospital or directly from the owners. Samples from Purdue had complete pathology including tumor grade and stage data, which confirmed that 92.3% of affected dogs had high grade (3-4) tumors at the time of diagnosis, and 44% had distant metastases at the time of death. Prior to the association analysis, we tested the samples for population substructure as well as correct breed assignment, disqualifying four dogs from breed-specific analyses and three dogs from all analyses (Fig. 1a and Supplementary Figs. 1 and 2). PCA showed clustering that appeared to correlate with affection status within the Sheltie cohort. One-way ANOVA confirmed that PC2 correlated with disease status (*P* = 0.0046, Fig. 1b). We therefore corrected for population substructure in both the within and across breed analyses.

The association analysis of 49 affected Shelties and 51 unaffected Shelties, aged 10 years and older (Table 1) revealed a single locus tagged by three SNPs, chr13: 44493602, 44508476, and 44520164, which was significantly associated with iUC (*P* = 1.05 × 10^−7^). The result remained significant after 10,000 permutations (P-perm_.05_ = 1.37 × 10^−6^) (Fig. 1c, Q-Q plot in Supplementary Figure 3). The GWAS was repeated after adding an additional 21 affected and 34 unaffected dogs from three related breeds: Collie, Border collie, and Australian shepherd (Fig. 1d). These breeds were chosen because they are closely related to Shetland sheepdogs^28^, share other deleterious mutations^29,30^ and we had a sufficient number of affected dogs on hand. The addition of other breeds that share association within the same region of the genome has been shown to greatly reduce the size of associated haplotypes by providing additional recombination events, thus improving the chance to find a shared disease associated mutation^29,31–33^. The same three SNPs were associated with iUC in the multi-breed cohort, *P* = 5.36 × 10^−10^ (Fig. 1e, Q-Q plot in Supplementary Figure 3).

**Table 1:** Samples included in GWAS analyses

	Affected			Unaffected		
Breed	Number	Age^1^ (range)	Sex (%F)	Number	Age^1^(range)	Sex (%F)
Shetland sheepdog	49	11 (5–16)	58%	51	12 (10–16)	57%
Collie	6	10 (6–11)	29%	12	4 (0.1–12)	50%
Border collie	12	10 (7–13)	73%	12	8 (1–14)	45%
Australian shepherd	4	10 (10)	100%	10	8 (3–12)	50%

^1^Age is given as the average across all samples in the group.

**Fig. 2:**
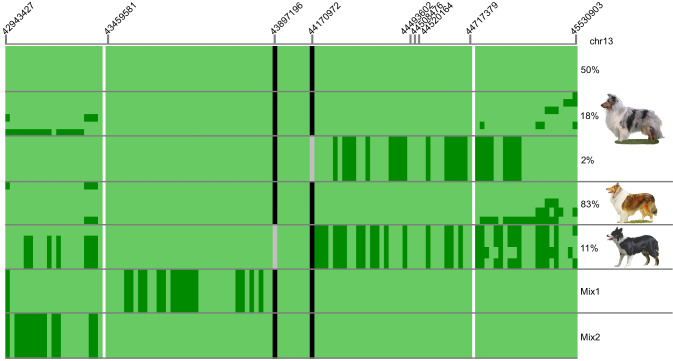
iUC associated haplotypes across the chr13 locus. The haplotypes comprise 120 markers represented by vertical bars. White vertical lines indicate the boundaries of the core haplotype. Alleles present in the Sheltie iUC associated haplotype are light green and the alternate allele is dark green. The putative causative variants are shaded black when present on the haplotype or grey when not present. The percent in affected dogs of each haplotype, or cluster of haplotypes sharing the same core, is listed on the left followed by the image of the breed top to bottom: Shetland sheepdog, Collie, Border collie. Mix 1 and 2 are atypical Border collies 1 and 2, respectively.

We calculated linkage disequilibrium (LD) from the peak SNP to all others on the chromosome and identified a region between base-pairs (bp) 42943427 and 45635207 that included markers with a pairwise r^2^ ≥ 0.6 to the peak SNPs and an association with iUC of *P* ≤ 1 × 10^−5^ (Fig. 2). This locus spans a region of significant dysregulation previously identified in an RNAseq analysis of multiple iUC tumors^34^(Supplementary Fig. 4). Genotypes were phased across the locus and haplotypes estimated. More than 25% of the affected Shelties were homozygous for a single haplotype across the entire 2.69 Mb region. A core haplotype was identified between two sites of predicted recombination (43459581 and 44717379) (Fig. 2) that was found in 40 of the 49 affected dogs, ~60% of which were homozygous. This haplotype was also the most common across all Shelties, accounting for 53% of total haplotypes: 68% in cases and 39% in controls, (OR 2.61, CI (1.01–6.66), chi^2^ *P* = 3.20 × 10^−15^). The core haplotype was also homozygous in five of six affected Collies.

### Genome sequence analysis

**Fig. 3:**
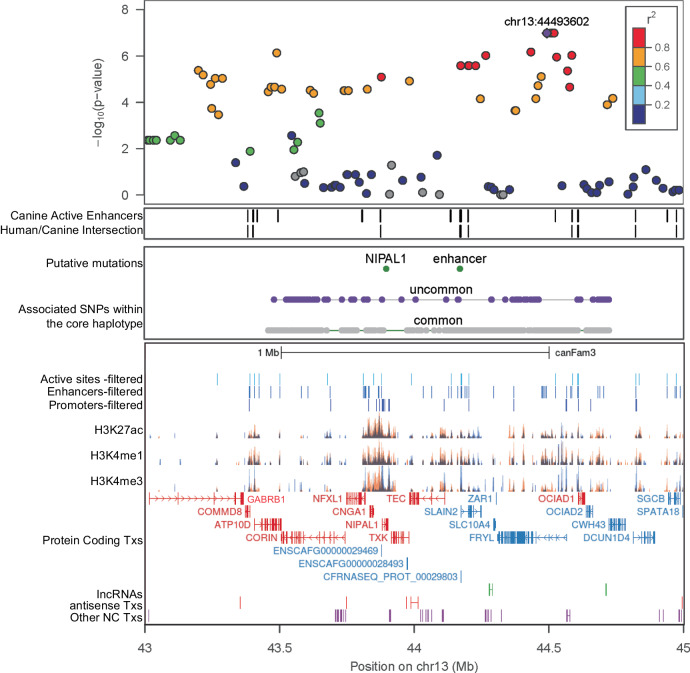
Detailed graph of the associated locus on chr13. LD between the top SNP and all others in the region is indicated by the color of the markers with red indicating highest value. Positions of predicted active regulatory regions are in the second panel. The third panel contains positions of the associated SNPs within the shared haplotype with the presumed functional SNP highlighted in green. The bottom panel contains regulatory regions from ChIPseq as well as gene annotations. Each gene is represented by the longest transcript from the Broad Improved Canine Annotation v.1 (UCSC). The components are plotted against the CanFam3.1 genome using custom tracks in the UCSC browser (http://genome.ucsc.edu). Genes highlighted in red were upregulated in iUC tumors^34^.

We sequenced the genomes of seven affected and five aged, unaffected Shelties (Supplementary Table 1). These were aligned to CanFam3.1 and UU_GSD1.0 reference sequences, and genotypes were called in conjunction with dogs from multiple, diverse breeds to test for possible causative mutations. The samples were chosen based on their haplotype across the chr13 associated locus. Using a combination of three canine gene annotation models, a list of curated enhancer regions derived from 131 human biosamples^35^, and ChIPseq data from two canine iUC cell lines, we assessed the single nucleotide variants (SNVs) on the core-associated haplotype for possible functionality (Fig. 3). There were 1968 variants that displayed the same allele distribution as the peak SNPs from the GWAS. Of these, 115 were in regulatory or non-coding elements and 47 were in protein coding transcripts. These could be further categorized into 27 variants in three-prime and five-prime UTRs, 12 synonymous variants, and eight missense variants (Supplementary Table 2, complete list can be found at https://research.nhgri.nih.gov/dog_genome/data_release/index.shtml). Four of the missense variants were in non-conserved regions of genes and therefore less likely to be damaging. The sites of the four remaining variants were highly conserved between dog and human in both genomic sequence and protein sequence therefore they were converted to human coordinates and assessed for potential pathogenicity using 11 different variant effect predictors. Only one of the coding mutations, a G > A missense mutation at position 43897196/CanFam3.1 (chr13:45186252/UU_GSD1.0), was predicted to be potentially pathogenic. It encoded a G256D change in the *NIPAL1* gene. This highly conserved amino acid is within the sixth transmembrane domain of the protein and is predicted to disrupt the helix structure (*P* = 6.0 × 10^−3^ MutPred2) (Supplementary Fig. 5). Excluding the breeds analyzed in this study, this mutation was found in only one dog from a data set of 1330 for which WGS was available, and in zero out of 1894 dogs published by the Dog10K consortium^36^.

Because our previous study showed that this region harbors numerous upregulated genes, including *NIPAL1*, we also evaluated putative regulatory variants. We compared the sites of known human enhancers to the active enhancer regions predicted from ChIP-seq in canine iUC cell lines (Fig. 3). We identified 19 variants that were within sites corresponding to active enhancers present in both species that, in humans, are predicted to affect multiple genes (Supplementary Table 2). Because markers across this region had identical genotypes in the 12 sequenced dogs, we assessed the frequencies of the iUC associated alleles in other dog breeds. Among the 1237 dogs and 93 wolves, twelve of the 19 iUC associated enhancer alleles were major alleles in the general population and another six had allele frequencies > 0.34 in dogs and > 0.24 in wolves. One variant, located at chr13:44170972, 1260 bp upstream of the *SLAIN2* gene, had an allele frequency of only 0.05 among dog breeds not in this study, and was not found in wolves. The change of alleles at this variant, chr13:44170972 G > A, results in the creation of a binding site for 14 different transcription factors or transcription factor complexes, as ascertained using two different prediction methods (Supplementary Table 3). Increased affinity for any of these transcription factors to the region could lead to dysregulation of local genes.

We sequenced the two mutations within the chr13 locus in 47 affected and 47 unaffected Shelties and 150 dogs from related breeds, including 34 with iUC. The missense mutation at chr13:43897916, which we refer to as NIPAL^G256D^, was found in 85% of the affected Shelties and was homozygous in 60%. The odds ratio for developing iUC with at least one copy of the mutation was 2.95 (CI 1.08–8.05), and if homozygous for the mutation, the OR increased to 8.42 (CI 3.12–22.71). The mutation within the putative multi-gene enhancer was found in 83% of affected Shelties and was homozygous in 57%. The OR for having the disease with at least one copy of the enhancer mutation was 2.76 (CI 1.05–7.26), and in the homozygous state, OR = 7.71 (CI 2.87–20.75). Recombination between these two variants was observed in only two Shelties, one affected and one unaffected; both carried NIPAL1^G256D^ but not the enhancer variant. These risk ratios are much higher than any described for variants associated with human bladder cancer.

The NIPAL^G256D^ variant was also found in seven affected Collies and two affected Border collies. The enhancer variant was found in the same nine dogs and in two additional affected Border collies. Neither variant was found in the other related breeds (Table 2).

**Table 2:** Genotypes of putative causative mutations within dogs of each breed

		43897196			44170972		
		AA	AG	GG	AA	AG	GG
Shetland sheepdog	Affected	28	12	7	27	12	8
	Un-Affected	7	24	16	7	23	17
	Gen Pop^1^	20	27	35	22	27	33
Collie	Affected	7	0	1	7	0	1
	Gen Pop^1^	5	13	14	4	14	14
	Gen Pop >10	0	1	1	0	1	1
Border collie	Affected^2^	0	0	11	0	2	9
	Gen Pop^1^	0	0	61	2	16	43
	Gen Pop >10	0	0	17	0	4	13
Sheltie Mix	Affected	0	2	0	0	2	0
Other related^3^	both	0	0	36	0	0	36

^1^Gen Pop or general population dogs are all ages and were not diagnosed with iUC at the time of collection. Gen Pop > 10 are general population dogs greater than 10 years old. ^2^Two of the affected Border collies were determined to be Shetland sheepdogs mixes reducing the Border collie affected counts. ^3^Other related breeds (13 affected and 23 unaffected) include Australian shepherd, Bearded collie, Pembroke Welsh corgi, Australian cattle dog.

We noted that the two affected Border collies who carried NIPAL1^G256D^ were highlighted in the population analyses as atypical Border collies because they did not cluster with the other members of the breed (Fig. 1a and Supplementary Figs. 1 & 2). To determine if these two dogs might be admixed, we phased their genomes and calculated haplotypes predicted to be identical-by-descent (IBD) with the dogs from 250 breeds in the 1330 dataset, plus the breeds from this study. Both dogs displayed the highest amount of IBD haplotype sharing with Shelties and a unique mixture of other breeds (Supplementary Fig. 6). Neither showed any Border collie ancestry beyond what would be expected for a Sheltie. In addition to the two mutations, both carried the Sheltie iUC-associated core haplotype. This haplotype was not found in any purebred Border collies. This data, therefore, identifies NIPAL1^G256D^ in Shelties, Collies and in affected mixed breed dogs with Sheltie ancestry.

Though NIPAL^G256D^ was not found in purebred Border collies, the enhancer variant was. We phased the chr13 locus in a total of 56 Border collies and genotyped the two iUC associated variants. We found 17 Border collies that carried the enhancer variant and none carried NIPAL^G256D^. Interestingly, all Border collies with the enhancer mutation carried a haplotype that matched the Sheltie iUC-associated haplotype between chr13:43453329 and 44174029, which is approximately half of the core haplotype including the sites of both putative disease-causing variants. This suggests NIPAL1^G256D^ appeared on a haplotype that carried the enhancer mutation, possibly after the division of the breeds in the late 1800s.

### Additional GWAS

The alleles on chr13 contribute to a substantial portion (16–41% in this dataset) of iUC cancer risk in Shelties. However, in a group of 87 healthy Shelties of all ages, some of which will likely develop cancer as they age, the NIPAL1^G256D^ allele frequency was 0.41, a larger percentage than we would expect for iUC. The difference between cases and controls lies in the frequency of homozygous mutations. Approximately 60% of the affected dogs are homozygous for the *NIPAL1* mutation compared to <15% of the unaffected dogs.

**Fig. 4:**
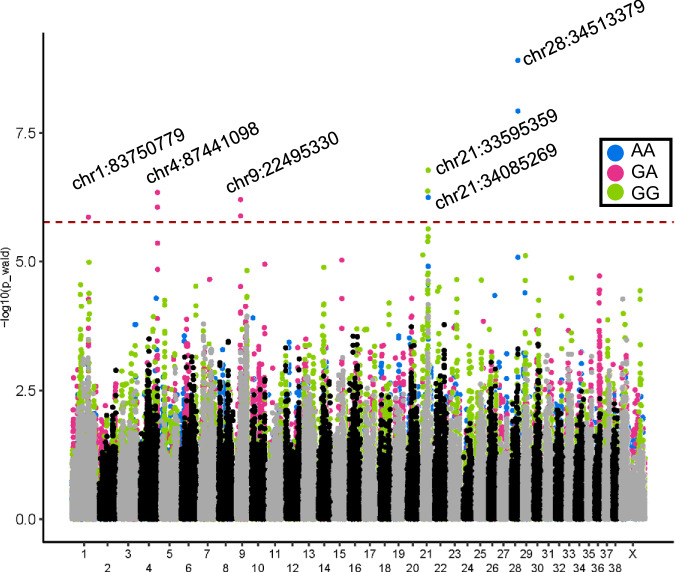
GWAS results from the Shetland sheepdog cohort divided by *NIPAL1* variant genotype. The results from three independent GWAS are combined for comparison. Each is colored by genotype at NIPAL1^G256D^ according to the key provided. The significance line is set at 1.7 × 10^−6^.

Though homozygosity plays a large role in the risk conferred by NIPAL1^G256D^, there are undeniably additional contributing loci. To locate genomic regions that moderate risk within the Shelties, we performed three additional GWAS, grouping dogs based on their genotype at chr13:43897196: all dogs that are homozygous for the mutation, those that are heterozygous, and those that do not carry the mutation (Table 2). Subsetting the dataset in this manner removes the contribution of the chr13 mutations, thus revealing additional risk loci. These three analyses, for which cases and controls have the same genotype at NIPAL1^G256D^, revealed six additional loci on five different chromosomes associated with iUC (*P* < 2.06 × 10^−6^) (Fig. 4).

Calculating LD and from the peak SNP at each locus produced associated regions ranging from 100 kbp to 2.5 Mbp (Supplementary Table 4). The loci on chr1 and 28 were gene poor. The locus on chr4 lies within an F-box gene, *FBXL7*, which plays a role in cell cycle control, and the locus surrounding chr21:34085269 comprises four protein coding genes, three of which have a role in cancer growth. This locus overlaps the associated locus surrounding chr21:33595359, but the two tagging markers, chr21:34085269 and 33595359, are not in LD (r2 = 0.047). The second locus spans more than two megabases and encompasses 30 genes. The locus surrounding chr9:22495330 encompasses 26 genes (Supplementary Table 4).

**Fig. 5:**
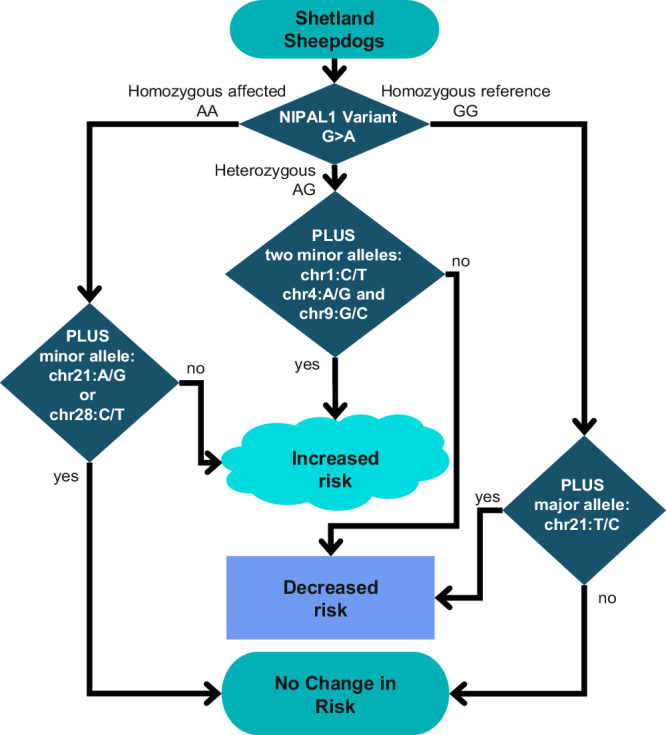
A flow chart of predicted risk for variant combinations leading to the development of iUC. Combinations of alleles at six loci increase the risk of or the protection from iUC in dogs carrying one of the three possible genotypes for NIPAL1G256D.

By calculating the OR for the multiple genotype combinations, we determined the effect each locus is likely to have on disease risk in Shelties (Fig. 5, Table 3). We find that homozygosity of the major allele for at least two of the chr1: 83750779, chr4: 87441098 or chr9: 22495330 loci offers a protective effect to individuals that are heterozygous for the NIPAL1^G256D^ variant, while the opposite, carrying the minor allele at two of the loci, increases the odds of developing iUC (Table 3). The other loci provide only risk or protection, not both. These combined data provide a platform for identifying interacting genes/mutations and common pathways that lead to cancer susceptibility in Shelties and are foundational in continued searches for iUC disease-associated mutations in other breeds.

**Table 3:** Odds ratios for disease risk given combinations of genotypes at the NIPAL1G256D and associated SNPs at six additional loci

NIPAL1G256D Genotype	AA		AG			GG			
Chr^1^	21 (1)	28	1	4	9	21 (2)	OR	CI	risk
Associated SNP genotypes	AA	**					18.5	3.1–22.7	increase
	**	CC					58.2	7.4–457.7	increase
			C*	A*	**				
			C*	**	G*		19.1	1.1–342.8	increase
			**	A*	G*				
			TT	GG	**				
			TT	**	CC		0.06	0.02–0.22	decrease
			**	GG	CC				
						T*	0.13	0.04–0.49	decrease
Disease associated allele frequency^2^	0.92	0.82	0.05	0.2	0.08	0.37			

^1^Chromosomes numbers indicate loci associated with iUC on each chromosome as described. 21(1) is chr21:34085269, 21(2) is chr21:33595359. ^2^Disease associated allele frequency calculated in 201 Shetland sheepdogs of all ages with no known cancer diagnosis. *OR*=Odds Ratio, *CI* = 95% confidence interval, *=any allele, **=any allele on both chromosomes.

### Somatic mutation

Two of the supporting loci, one at chr9:22381987-23201516 and the other on chr21: 31405230-33951574 include the oncogene *ERBB2* and a tumor suppressor *WEE1*, respectively. These genes are frequently mutated and/or dysregulated in human bladder cancers. We did not find germline mutations in the coding regions of either *WEE1* or *ERBB2* in the WGS of seven Sheltie cases. However, when we analyzed the sequence of these two genes in three canine iUC whole tumor sequences and 15 previously published whole transcript sequences, we found somatic mutations in each gene. Specifically, after applying multiple variant effect predicting programs, the *ERBB2* gene had two putatively damaging missense variants, chr9:22775561 C > A and chr9:22775767 C > G, each found in two tumor alignments, which create the D277Y and D251H protein changes in *ERBB2*. There was also a single putatively damaging C > G variant in the *WEE1* gene at chr21:32843843, leading to a predicted P175R protein change (Supplementary Table 5). A second predicted damaging variant at chr21:32854760, V468A within the kinase domain of *WEE1*, was identified in another tumor however evidence from RNAseq was not sufficient to rule out error and further sequencing is required to confirm. An additional mutation affecting ERBB2, G292R, was found in one canine transcript. This germline mutation creates a G292S change in humans which has slightly reduced effect predictions than the dog variant. Each *WEE1* variant was found in a single tumor while the *ERBB2* variants were each found in two different tumors for a minimum putatively damaging gene mutation frequency of 6% and 24% respectively. Additional coding variants with benign predictions were found in each of these genes.

## Discussion

**Fig. 6:**
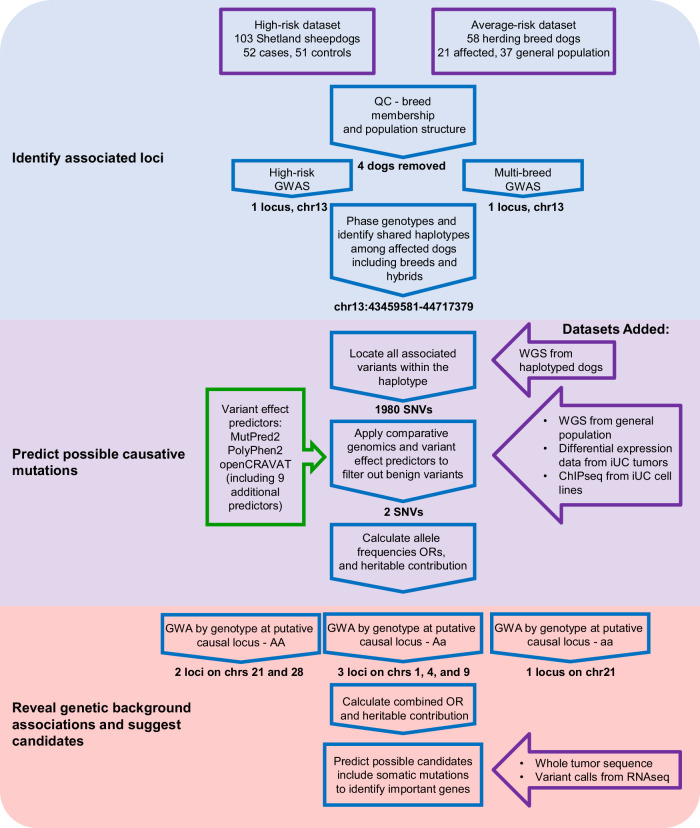
The order of analyses employed in this study. These steps were taken to identify possible genetic variants predisposing dogs to iUC and candidate genes that may play an important role in promoting or preventing disease development. Analysis stages are indicated by changing background color. Datasets used are listed inside purple shapes and predictive software in green.

There are over 350 recognized dog breeds in the world, each a unique population whose members share common ancestors, traits, and disease susceptibilities. Often multiple breeds will develop from a common working population due to geographic separation or regional preferences in size, coat color or working style. The Collie, Border collie, and Australian shepherd are closely related to the Sheltie and are all descended from the original working collies of the British Isles, prized for their ability to herd sheep. Overall these farm dogs have given rise to least ten independent yet related dog breeds^28^ that share disease risk associated genetic variants^29,30,37^. In this study (Fig. 6) we examined susceptibility to iUC in the above four breeds, one of which, the Sheltie, demonstrates a particularly high disease risk^12^. We observe that the Sheltie and Collie carry the same disease-associated coding variant NIPAL1^G256D^ and, along with the Border collie, share a putative regulatory variant associated with iUC located 273 kb downstream. These variants may also exist at low frequency in additional related or descendant breeds.

Dog breeds are developed by reproductively isolating a small group of individuals from the general dog population, therefore we can treat each breed as a large extended family, rather than a geographic or ethnic population, and assume a common risk variant will be responsible for the majority of disease in any given breed^38,39^. In essence, the number of breeds or lineages is the major determinant of power, and not the number of individual dogs. In a high-risk breed, the disease associated variant is often found at high frequency and can be used to further subset the population into genotype-specific clusters to find additional disease loci. Secondary loci may modify the effect of the primary locus or may themselves be primary risk loci that only exist in a small subset of the breed, perhaps in the presence of a particular aspect of the genetic background. We have taken advantage of breed structure to identify a total of seven iUC-associated loci by segregating samples based on their genotype at an initial, primary locus, *NIPAL1*. Although the additional loci likely play only secondary roles in Sheltie iUC risk, they may be part of the primary path to disease development in other breeds. Within the Sheltie iUC-associated loci we identified germline or somatic variants in three genes that, combined with the previously identified BRAF^V595E^ somatic mutation, point towards the disruption of the MAPK signaling pathway and its components as a likely mechanism for iUC development in dogs.

The most compelling mutation found in the strongly associated chr13 locus is a variant within the *NIPAL1* gene that causes the non-polar glycine at the 256^th^ position to be replaced with a negatively charged aspartic acid. The G256D mutation lies within one of the transmembrane domains of the NIPAL1 protein and is predicted to disrupt the helical structure, possibly by changing the steric configuration through size or charge. The NIPAL1 protein is a transporter of multiple divalent cations, most notably magnesium (Mg(2 + )). Magnesium levels and the disruption of magnesium transporters have been associated with cell proliferation, metastasis, and angiogenesis as well as effectiveness and toxicity of chemotherapeutic treatments in bladder and other cancers^40,41^. In human bladder cancer cell lines, 24 h treatment with MgCl_2_ is shown to alter the expression and phosphorylation of multiple components of the MAPK signaling pathway compared to untreated cells^42^. The pathway is crucial for tumor development and is frequently dysregulated in both human (reviewed in refs. ^21,43^), and dog bladder cancers^20,44^. The mutation is expected to alter the ability of the gene to transport magnesium, and possibly other ions, across cellular membranes, thus affecting magnesium concentration within cells.

Interestingly, *NIPAL1* is also associated with gout in a GWAS of more than 4000 Japanese men^45^. Gout is a form of hyperuricemia that can occur in the presence of reduced renal urate excretion, and affected individuals have a 1.18 fold increased risk of developing bladder cancer (95% CI = 1.05–1.32)^46^. Uric acid levels are significantly increased in the urine of dogs with bladder cancer compared to matched controls^47^, and while this relationship has been reported in multiple studies, the underlying cause remains unknown^48^. *NIPAL1* may also play a role in regulating the transport kinetics of urea in bladder cells^49^.

In addition to the above, we identified a putative enhancer variant at chr13:44170972 that is in near perfect LD with the *NIPAL1* mutation. Based on synteny with human chr4:47547066-48980084 (hg38), this enhancer may affect the expression of 10 genes in the region, including *NIPAL1*^35^. Multiple studies show that *NIPAL1* is overexpressed in canine iUC tumors^34,50,51^, as are14 other genes within the primary risk-associated chr13 locus^34^. The enhancer variant alters putative transcription factor binding sites at the locus, creating new sites that bind AP-1 complexes, which are heterodimers of the JUN (Jun proto-oncogene AP-1 transcription factor subunit) and FOS (Fos proto-oncogene AP-1 transcription factor subunit) protein families. These complexes are frequently linked to tumorigenesis, potentially accounting for increased expression across the locus^52^. The MAPK signaling pathway regulates AP-1 through activation and by controlling expression^53^.

Secondary loci found on chr9:22381987-23201516 and chr21:31405230-33951574 comprise many potential cancer genes, but two, *Erb-B2 receptor tyrosine kinase 2* (*ERBB2*) and *WEE1 G2 checkpoint kinase* (*WEE1*), stand out as likely candidates for disease risk, as both are frequently mutated, amplified or dysregulated in human bladder tumors. *ERBB2* is a member of the epidermal growth factor receptor family and functions upstream of signaling pathways frequently implicated in cancer, such as the MAPK or the mechanistic target of rapamycin (mTOR) signaling pathways. Multiple studies of canine iUC tumors show that *ERBB2* is over expressed in canine bladder tumors compared to normal bladder tissues^34,51,54^. We did not find any *ERBB2* disease-associated germline coding mutations in the WGS from seven Sheltie cases; however, we did find potentially pathogenic somatic alterations in whole genome sequence and RNAseq from iUC tumors. Mutations at chr9:22775767 and chr9:22775561 correspond to the protein alterations D251H and D277Y in ERBB2, respectively. Both variants are predicted to be damaging by multiple metrics (Supplementary Table 5). Twelve percent of human bladder tumors have mutations in *ERBB2*, and the D277Y protein variant is among them^55^. Though the D251H variant has not yet been found in human cancers, multiple mutations in the adjacent amino acid at position 250 have been reported. An exome study of shed DNA from dogs with suspected bladder cancer also found the nearby ERBB2 mutation, G292R^20^ which was observed in one of our transcript sequences. These variants are found in the furin-like domain of the protein which includes the most common human somatic variant in the gene, S310F. In aggregate this argues that *ERBB2* plays an important role in canine iUC disease risk.

An additional gene implicated in human bladder cancer, the tumor suppressor *WEE1*, is located in the disease associated chr21:31405230-33951574 region. *WEE1* regulates the G2/M checkpoint through an activation loop with cell division kinases, CDK1 and CDK2, within the MAPK signaling pathway^56,57^. Activating BRAF mutations, which are found in the majority of canine iUC tumors, increase cellular levels of WEE1 protein^58^. Through phosphorylation of the CDKs, *WEE1* plays a role in homologous recombination DNA repair, and because cell cycle and DNA damage repair pathways are frequently interrupted during tumor development, *WEE1* inhibitors are effective cancer therapeutics^59,60^. We again identified at least one somatic mutation, P175R, in an iUC tumor. This mutation is in the N-terminal regulatory domain of WEE1, adjacent to the nuclear export signal and is predicted to affect the function of the gene. In humans bladder urothelial carcinomas *WEE1* is frequently dysregulated through copy number loss (13.7%)^57^.

Combined, these findings point to repeated disruption of the cellular proliferation and DNA damage repair pathways as mechanisms for development of iUC in dogs. Our data suggests that increased risk is caused through germline variants that provide the initial step in the cascade. We have identified one common germline mutation (NIPAL1^D256G^), disease associated sites in the regions of *ERBB2* and *WEE1*, and the presence of somatic mutation in the same genes suggesting they are important in canine iUC progression. Combined with the previously identified BRAF^V595E^ mutation, our findings point toward the MAPK signaling pathway and its components as a key driver of iUC in dogs.

By combining genotypes from seven associated loci we can account for the development of iUC in 75% of Shelties. These predictions could improve after analysis of a larger cohort. For example, there were no unaffected dogs heterozygous for the *NIPAL1* variant that carry the minor allele at the chr1 locus. Similarly, only affected dogs that do not carry the *NIPAL1* variant are homozygous for the alternate allele at chr21:33595359. To calculate the odds ratios, we assume a frequency of >0 for these combinations. If these combinations are not seen at increased numbers, they would account for disease risk in an additional 10% of cases, bringing the ability to predict disease as high as 85%. While this would make an excellent start in genetic testing for iUC risk in Shelties, these findings need to be verified in an unrelated set of dogs.

There are factors that limit the generalizability of these results: limited sample sizes and a lack of direct functional assessments for the putative mutations. The modest sample size is appropriate for finding variants of moderate to large effect in dogs given their population structure, but may lead to false negatives. We are unlikely to recognize variants with very small effect sizes given the small number of individuals included in the GWAS and the WGS was targeted specifically for finding high-effect variants on chr13. It is also possible that there are low penetrance variants contributing to the disease that are fixed within this population. We expect these low-effect and low-penetrance variants to reveal themselves as additional high-risk breeds are analyzed and datasets are combined. The dog genome project has advanced substantially within the last decade with the addition of four new reference sequences and 19 new assemblies (NCBI). There is also a finely filtered and vetted variant dataset of over 2000 whole genome sequences^36^. However, there remains a dirth of experimentally confirmed protein function data, thus comparative genomics provides the majority of data regarding gene function^61–63^. This is not necessarily a problem, however, as comparative genomics between dog and human features numerous successful studies that identify altered genes that share function in the same disorders in both species (reviewed in^64–66^. The next step in our studies will be to perform functional analyses on the newly identified and predicted pathogenic mutations using protein assays to determine specific activity, allele-specific functional studies in cell cultures, or by creating mouse models. There are also non-coding RNAs in the dog genome that have yet to be annotated due to a lack of sequence conservation. For example, there is a lncRNA, *OCAID1-AS1* that has been linked to bladder cancer prognoses in humans^67^ within our primary associated region on chr13. This lncRNA is weakly conserved and not annotated in dogs and though there are no associated mutations in the corresponding locus, we may be missing changes in expression that correlate with tumor behavior.

Finally, we note that the study of iUC in canine populations may fill in gaps in studies of bladder cancer in humans. The numerous cohorts amassed for studies of human bladder cancer risk feature, on average, > 80% males and 80–85% non-muscle invasive disease^68–71^, even though muscle invasive bladder cancer carries a much higher risk of early mortality^72^. Women are underrepresented in these studies because they are less likely to get bladder cancer than men, however they more often get invasive disease and their relative survival expectancy is reduced by five percentage points or more across all stages of the disease (https://seer.cancer.gov/). In dogs, females are either equally susceptible or more likely to get iUC than males, depending on the breed. Our dataset included 59% female and 41% male affected dogs and >90% invasive disease. Because of these factors, the dog is likely to provide new information about genes and pathways important to a highly destructive subset of bladder cancers in humans.

## Methods

### Sample collection and DNA extraction

Affected and unaffected dogs from the high-risk breed were solicited through the Purdue University Veterinary hospital as well as through dog clubs and at dog events. Whole blood samples were collected from dogs with iUC through either the Purdue University Veterinary Hospital, where they had biopsy-confirmed iUC, or via direct mail from owners of affected dogs. Owners of dogs not diagnosed at Purdue submitted pathology reports with diagnosis from the treating veterinarian. Affected dogs had no reports of other cancers. Unaffected dogs were ≥ 10 years old at the time of sampling with no reports of any cancer. Both groups were filtered for relatedness so that no two dogs shared grandparents and they were matched for sex. Affected dogs from average risk breeds were collected through the Purdue University Veterinary hospital and unaffected dogs were chosen from population studies (Table 1). Similar human studies often recruit from high-risk clinics, standardized screening programs, or make use of data from HMOs or insurance companies. However, few such programs exist in the veterinary world, e.g., there are no pet HMOs and pet insurance is still in its infancy. However, the very nature of being a Shetland sheepdog puts dogs of this breed in a high-risk category. Further, samples were collected from Purdue Veterinary hospital department of oncology, which specializes in the treatment of urinary carcinoma, thus insuring a high-quality dataset. Collection was performed according to the NHGRI Animal Care and Use committee approved protocol GFS-05-1 or the Purdue Institutional Animal Care and Use committee approved protocol 1111000169. Dog owners signed informed consent documents prior to collection. DNA was extracted from whole blood samples using cell lysis followed by phenol-chloroform, as described previously^73^, with phase separation performed in 15 mL Phase Lock tubes (5-Prime, Inc. Gaithersburg, MD, USA). DNA was resuspended in a 10 mMolar (mM) tris, 0.1 mM EDTA solution and stored at −80 ^o^C.

### Sample filtering

In order to include non-pedigreed dogs, all samples were assessed for breed membership by combining the cancer genotyping data with previously published population dataset including 157 breeds in addition to the iUC study breeds^28^, and using principal components analysis (PCA) and distance measurements calculated in PLINK v.1.90^74^. The all-breed data can be obtained at https://research.nhgri.nih.gov/dog_genome/data_release/ or through the NCBI Gene Expression Omnibus (GEO) data repository under accession numbers: GSE90441, GSE83160, GSE70454, and GSE96736. Significant PCs were determined using the Tracy-Widom distribution^75^. The first three PCs significantly separated the four UK herding breeds: Shetland sheepdog, Collie, Border collie, and Australian shepherd (Fig. 1 and Supplementary Fig. 1). Samples that did not cluster with their assigned breed on PCA were marked for follow-up. Samples were then added to a neighbor joining tree which included an additional 157 previously published breeds^28^ using a 1-ibs distance matrix calculated in PLINK. Of the samples marked for follow-up after PCA, three putative Shetland sheepdogs did not cluster with the pedigreed Shetland sheepdogs on the multi-breed tree and were therefore excluded from the Shetland sheepdog analysis. Samples that did not cluster with any of the UK herding breeds (two of the above Shetland sheepdogs and one Australian shepherd) were excluded from all analyses. Samples that clustered with the UK herding breeds, but not with their assigned breed, were retained for the multi-breed herding dog analyses (one Australian shepherd, two Border collies and one Shetland sheepdog). All other samples clustered with the submitted breed in both analyses (Supplementary Fig. 2).

The ancestry of the four herding breed dogs, designated atypical, was determined by calculating shared haplotypes, estimated to be identical by decent (IBD) using the BEAGLE v4.1 ibd=true option. Total shared haplotypes were summed between all pairs of dogs as described previously^28^ this time comparing breed dogs to the unknown dog rather than breed to breed. Breeds with average total haplotype sharing with the unknown dog above the 95% percentile of all comparisons were considered possible contributors. Final relationships were visualized using Circos in R^76^(Supplementary Fig. 6).

### Single Nucleotide Polymorphism (SNP) genotyping and GWAS

DNA samples from 103 Shelties and 58 dogs from related herding breeds were genotyped at 170k loci using the Illumina CanineHD bead chip with standard protocols (Illumina, San Diego, CA). Genotypes were called in Genome Studio 2.0.4 and exported using the CanFam3.1 forward strand orientation. Data was imported into pLINK v1.9 for filtering and reformatting. Single nucleotide polymorphisms (SNPs) missing more than 10% of genotypes, or with minor allele frequencies (MAF) < 5% in Shetland sheepdogs, were removed from the analyses. After filtering the final dataset included 90,530 SNPs and 155 dogs. Power analysis was carried out using genpwr^77^ in R (Supplementary Fig. 7).

Genomic inflation was calculated from the *p*-values from uncorrected association analysis in R with λgc > 1.1, indicating genome-wide inflation likely due to confounding population structure. PCA was run on the dataset and one-way ANOVA test was calculated in R for each significant PC to identify stratification by disease. To correct for the population structure, GWAS was performed in GEMMA^78^ using DNA from 49 Sheltie cases and 51 aged ( ≥ 10 yrs) Sheltie controls. A second GWAS included additional DNA samples from 21 affected and 34 unaffected dogs from three related herding breeds (Collie, Border collie and Australian shepherd) (Table 1). Correction for multiple testing was applied in two ways; Bonferroni correction based on independent loci (pair-wise r^2^ < 0.80) P-Bonferroni_.05_ = 1.7 × 10^−6^, and by permuting the phenotypes 10,000 times and identifying the limit at which 95% of random associations could be excluded (P-perm 0.05 = 1.32e10^−6^).

Associated regions were defined as the area encompassing SNPs in LD (r^2^ > 0.6) with the most associated SNP and associated with the disease with a *P* ≤ 1 x 10^−5^. All SNPs within this region were phased using PHASE v2.1 with recombination^79^. The common Sheltie case haplotype was included as a known haplotype when phasing non-risk breed data.

Additional association analyses were performed on subsets of samples based on their genotype at chr13:43897196. The three sets include 29 cases and seven controls that were homozygous for the *NIPAL1*^*G256D*^ mutation, 11 cases and 31 controls all heterozygous for the mutation, and eight cases and 20 controls, none of which carry the mutation. The association analysis was also repeated in the full set of 101 Shetland sheepdog samples using the number of alternate alleles at chr13:43897196, the variant within the *NIPAL1* gene, as a covariate. All analyses were corrected for underlying population structure as described above.

### Whole genome sequence (WGS)

Twelve Shelties were chosen for further genotyping because of the haplotype they carried across the chr13 associated locus. We chose six samples that were homozygous for the disease associated haplotype, five with one copy of the disease associated haplotype and one that was homozygous for the most common non-disease associated haplotype. Germline whole genome sequencing of these 12 samples was carried out at the NIH Intramural Sequencing Center (NISC) using the Illumina TruSeq DNA PCR-Free Protocol (Illumina, San Diego, CA, USA) on an Illumina Novaseq6000. These data are available in the NCBI Short Read Archive (SRA) under BioProject numbers PRJNA448733 and PRJNA685036 (Supplementary Table 1). Paired-end data was aligned to the CanFam3.1 reference genome and variants called using GATK as described previously^80,81^. Annotation was applied to the variant sets using snpEff v5.0^82^ with the Ensembl gene set CanFam3.1.99 and also using VEP^83^ with the CanFam3.1plus gene annotation^84^. Polymorphic variants from the associated region were extracted using bcftools view command^85^. The alignment was repeated using the reference build UU_GSD1.0^86^ and the corresponding annotation to look for missing exons.

In addition to the 12 germline sequences described above, three canine iUC tumors were sequenced at 60x coverage and aligned to CanFam3.1. Somatic variants were called using Mutect2^87^ with both matched normal sequence and a population-based panel of common variants to rule out germline polymorphism. In addition, RNAseq data published previously^34^ was analyzed for somatic mutation. Bam files were prepped following GATK best practices workflow for RNAseq short variant discovery. Variants were called using Mutect2 with matched normal RNAseq or a panel of normal RNAseq if no matched normal was available. A population-based panel of variants was used to filter out common polymorphism. All variants within the genes of interest were confirmed manually in IGV^88^.

### Mutation identification

Variants identified in the 12 WGSs that followed the same pattern as the most significantly associated SNPs identified in the GWAS and appearing on the disease associated haplotype were retained for further investigation. The position of each variant in relation to annotated genes was assessed using SNPeff and the CanFam3.1.99 gene model, VEP with the CanFam3.1plus gene model^84^, and by overlap using a bed file of exon positions from the Broad canine improved annotation v.1^89^. Variants within coding transcripts predicted to alter amino acids were converted to human genome positions using lift-over (UCSC, hg19)^90,91^. Within coding sequences, only sites for which both the DNA sequence and predicted amino acid were conserved between dogs and humans were considered for further evaluation. To confirm that no coding variants were missing due to gaps in the reference sequence we realigned the sequence reads to the UU_GSD1.0 reference sequence^92^ and searched for variation within the updated gene annotation. Pathogenicity of variants was predicted using openCRAVAT^93,94^, which encompasses multiple predictive tools, MutPred2^95^, and PolyPhen2^96^.

Disease-associated non-coding mutations were filtered for overlap with sites of human enhancers that had confirmed local gene interactions^35^, and with predicted canine enhancers and promoters determined from ChIP-seq analysis of canine iUC tumor cell lines. The human enhancer loci were lifted to the dog assembly using UCSC lift-over^90^. The allele frequency of each variant was determined using a set of 1330 dogs representing >200 breeds and 139 wild canids, none of which had a known diagnosis of iUC. Variants were considered potentially disease contributing if they were within a canine active enhancer, in the corresponding site of a human cis-active enhancer, and had an allele frequency of <10% in unaffected, average risk breeds.

Transcription factor binding sites (TFBS) were identified in regions of interest using TFBind and AIModules. Significant matches were identified using an empirically determined cut-off value for sequence similarity based on position weighted matrices from TRANSFAC R.3.4 database implemented by TFBind^97^, or an odds ratio greater than seven and an odds ratio deficit less than eight, compared to the canonical sequence, as determined by similarity calculations from position weighted matrices from the JASPAR 2022 database for the AIModule predictions^98^.

### Chromatin immunoprecipitation followed by sequencing (ChIP-seq)

ChIP-seq was performed as described in^99^ from canine iUC cell lines K9TCC_Mx and K9TCC_Ab, which were provided by the Knapp lab at the Werling Comparative Oncology Research Center at Purdue University, College of Veterinary Medicine^100,101^. The cell lines were grown to 70–80% confluency in DMEM/F12 media as previously described^100,101^. Approximately 5 x 10^7^–1 x 10^8^ cells were used in each assay. Cells were cross-linked using 1/10 volume of 11% formaldehyde quenched with 1/20 volume of 1 M glycine after 10 min. Cells were washed twice with 1x PBS and harvested using a Cell Lifter (Costar #3008) in PBS. Cells were pelleted and lysed in three stages with a protease inhibitor cocktail (Roche #11873580001) added at each step. DNA in pelleted nuclei was sheared on ice using a GEX130 Ultrasonic processor (Sonics, Newtown, CT) in 3 ml volumes with the following settings: power set at 30% output, 25 s ON, then 60 s OFF, repeated for a total of eight minutes. Magnetic beads (Invitrogen Dynabeads Protein G, Thermo Fisher Scientific, Waltham, MA), previously bound with an antibody to one of the histone modifications, were washed with phosphate-buffered saline solution (PBS), resuspended, then added to the fragmented DNA on ice and incubated at 4 ^o^C overnight. Beads with bound DNA strands were washed five times, then rinsed with PBS. The final rinse was aspirated and 115 µl of elution buffer added. DNA was eluted at 65 ^o^C for 15 min, vortexing every two minutes, followed by overnight incubation on a Thermomixer R (Eppendorf, Enfield, CT). DNA was purified with phenol-chloroform after treatment with RNaseA and proteinase K.

Immunoprecipitation (IP) was repeated using 10 ug of Abcam (Cambridge, United Kingdom) antibodies to H3K4me1 (rabbit polyclonal-AbCam, #ab8895), H3K4me3 (rabbit polyclonal-AbCam, #ab8580), H3K27ac (rabbit polyclonal AbCam #ab4729) and one negative control (IgG rabbit, AbCam#46540). A 100 µl input control sample was taken from shorn DNA prior to each IP and sequenced to control for background signal in the IP. DNA was quantified using a Qubit fluorometer (ThermoFisher, Waltham, MA, USA).

The purified, IP DNA was submitted for sequencing at NISC, where paired-end libraries were made with inserts averaging 320 bps and run on an HiSeq2000 Sequencing System (Illumina) with 100 bp reads. The reads were aligned to the CanFam3.1 reference sequence using BWA-MEM^102^ and converted to bam files using samtools 1.10^85^. Alignments were filtered as described previously in ref. ^103^ and histone binding sites called by counting the number of mapped reads overlapping the peak using MACS2 2.2.1^104^, and the spatial clustering approach from SICER2^105^. Only those that were present in two different canine iUC cell lines, overlapping each other by at least 50%, and called by both SICER and Macs2, were retained for comparisons.

### Targeted genotyping

Primers were designed to amplify the region around the two variants of interest on chr13, chr13:43897196 (F- GCTCCCAAGAAGGGACAGAC; R- TTGGAATAAGATGACAGAGCAAG) and chr13:44170972 (F- AAACGATCCAGAGAGCAGATTAC; R- GAGCCCAGGCCAAGAGTG) using Primer3^106^. PCR was carried out on a SimplyAmp thermocycler or GeneAmp PCR system 9700 (Applied Biosystems, Foster City, CA) using AmpliTaq Gold (Thermo Fisher, Waltham, MA) using standard reaction protocols with a touch-down thermocycler program starting at 65 ^o^C annealing temperature and decreasing 0.5 ^o^C each cycle for 20 cycles then an additional 20 cycles at 55 ^o^C annealing temperature. Denaturing and extending temperatures were constant as per standard protocols.

Amplified DNA was sequenced using BigDye Terminator 3.1 (Applied Biosystems, Foster City, CA) and run on a 3730xl sequence analyzer (Applied Biosystems). The variants were genotyped using Sequencher 5.4.6 (GeneCodes Corporation, Ann Arbor, MI). Odds ratios were calculated using genotypes from 47 affected and 47 unaffected Shelties.

### Reporting summary

Further information on research design is available in the Nature Research Reporting Summary linked to this article.

### Supplementary information

Supplementary Figures and Tables

Reporting summary

## Data Availability

The datasets presented in this study can be found in public online repositories. Specifically: Illumina SNP chip data and ChIPseq data from cell lines has been submitted to the NCBI GEO database under accession GSE241367 and GSE254079 respectively. Whole tumor sequence is in the NCBI short read archive (SRA) under BioProject PRJNA1007700. Whole genome sequence can be found in the SRA under BioProjects PRJNA288568 and PRJNA685036. RNAseq was previously submitted to the SRA under BioProjects PRJNA559406 and PRJNA308949.

## Code availability

Code generated to support primary data analysis and build figures is available upon request. Software, versions, and associated parameters used can be found in the methods section.
