# Supplementary material for: Genome-wide analyses reveals an association between invasive urothelial carcinoma in the Shetland sheepdog and NIPAL1
Source: NPJ Precis Oncol. 2024 May 22;8:112. doi: 10.1038/s41698-024-00591-0 (PMC11111773; doi:10.1038/s41698-024-00591-0)
Supplement: Supplementary file 1 — Supplementary Figures and Tables [file 41698_2024_591_MOESM1_ESM.pdf]

Supplementary Figure 1.
A series of two-dimensional PC plots of data in Figure 1A. The three hybrid samples are circled. AUSS=Australian shepherd, BORD=Border collie, COLL=Collie, SSHP=Shetland sheepdog

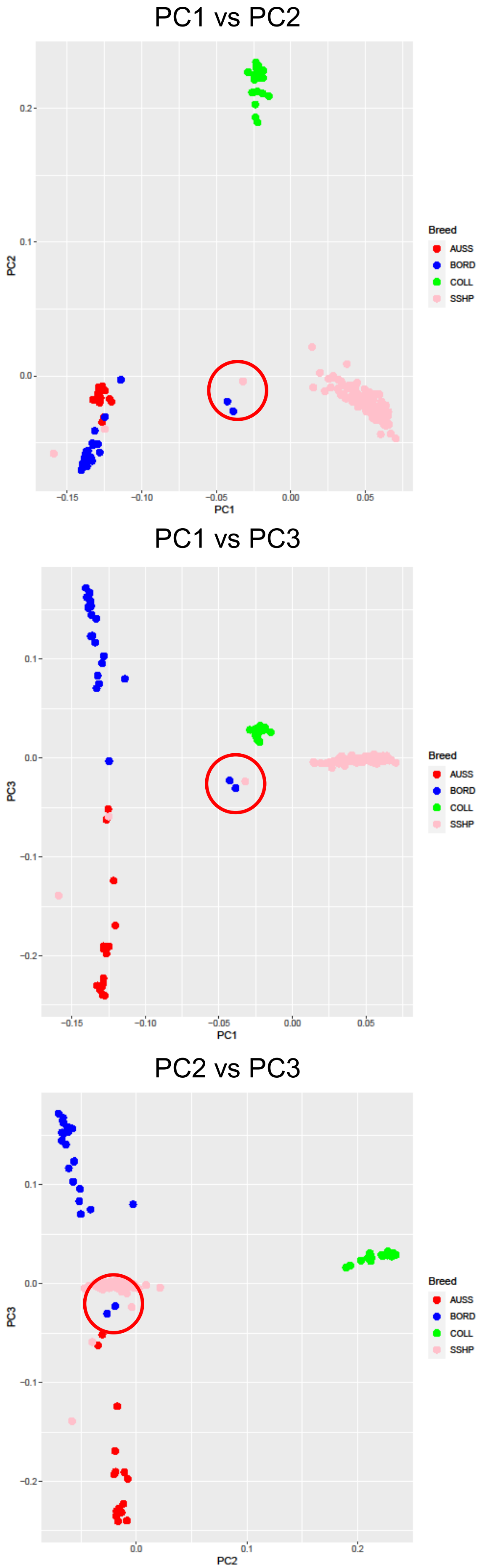

Supplementary Figure 2. Cladogram of dogs showing clutering by breed. Shown is the UK herding clade with the dogs included in the GWAS shaded dark green and pure-breed samples shaded light green. Affected dogs are marked with a dot near the branch. Blue stars indicated atypical examples of the reported breed from top to bottom: Shetland sheepdog 1, Border collie 1, Border collie 2, Australian shepherd 1. Grey stars represent dogs that were removed from the analysis as they are not the reported breed nor closely related.

Supplementary Figure 2

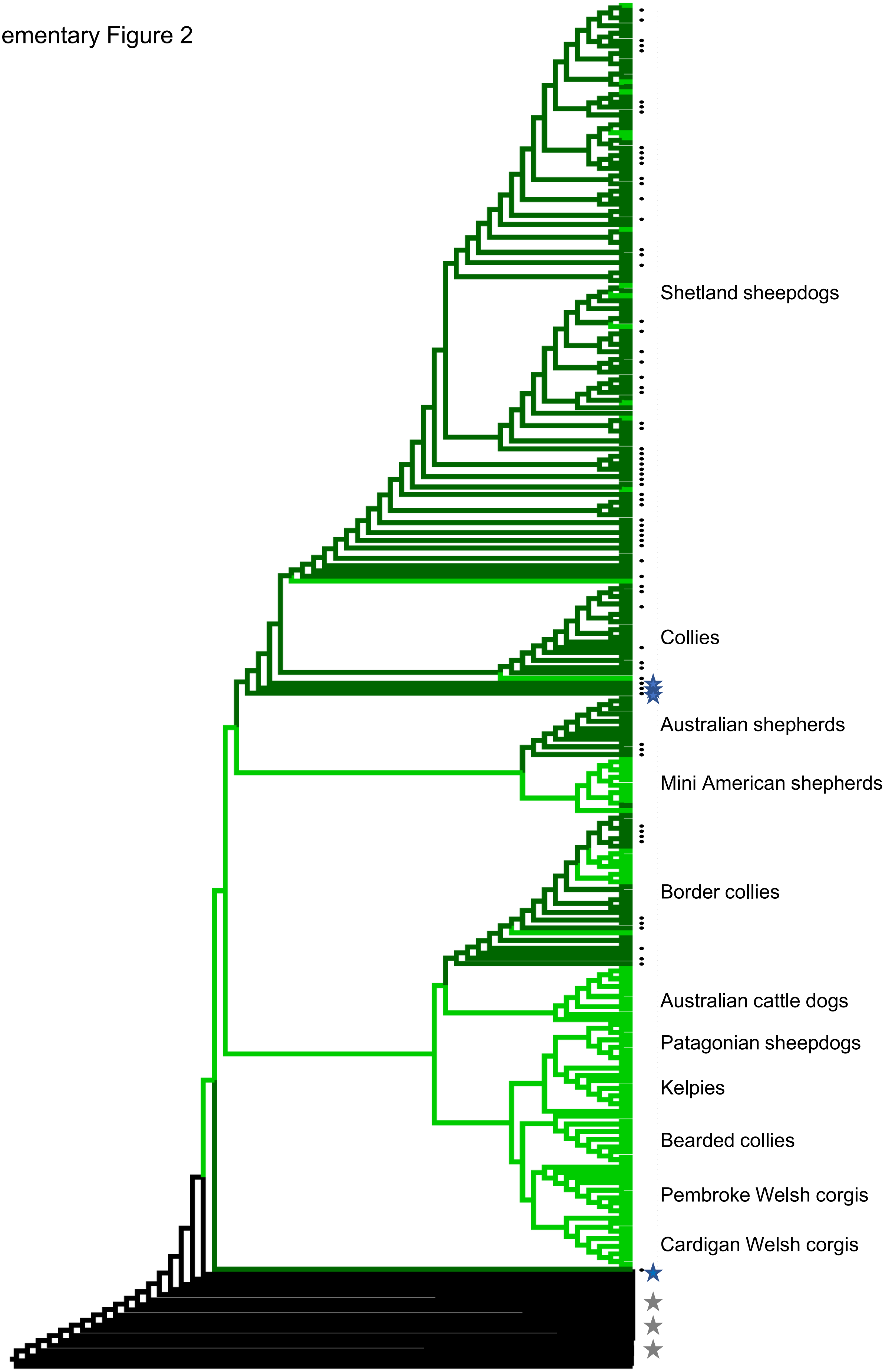

Supplementary Figure 3. QQ plots corresponding to the GWAS results in Figure 1.

a.

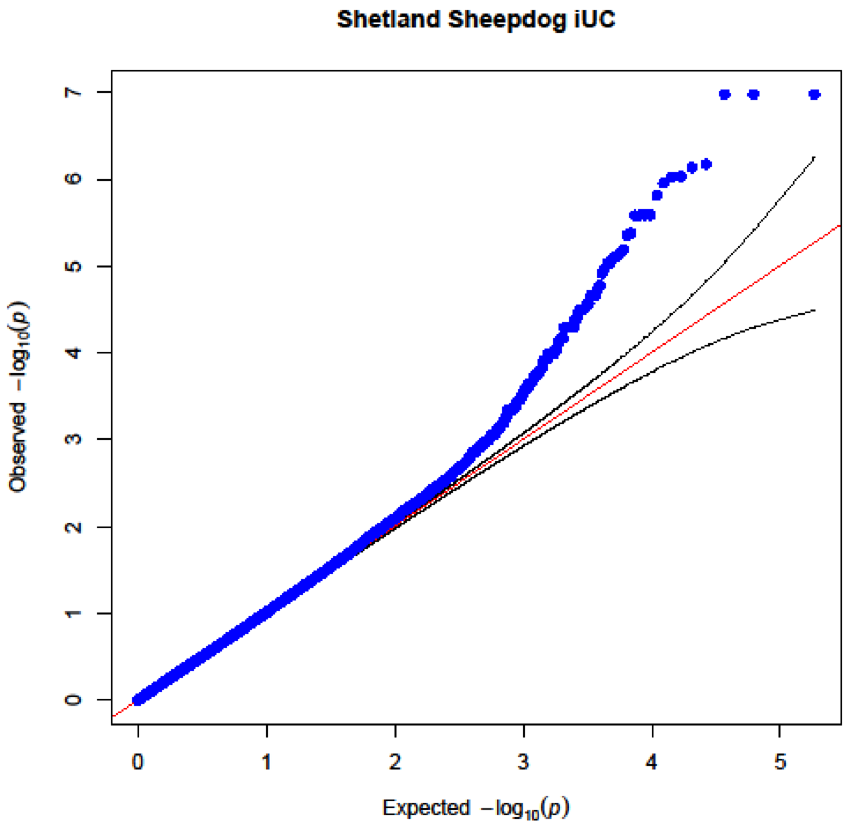

b.

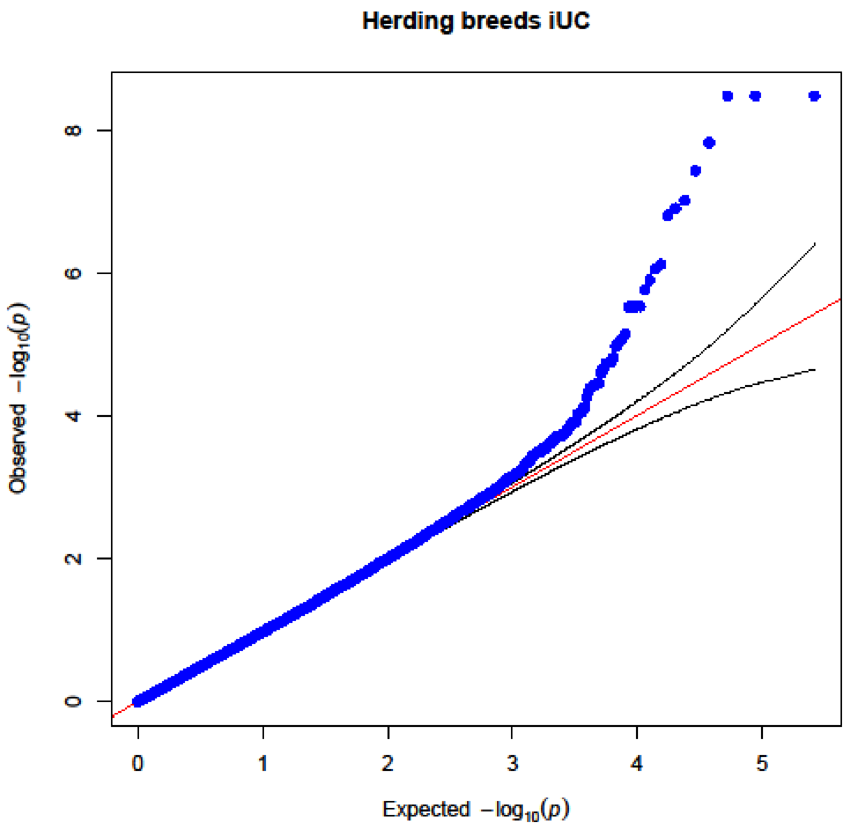

Supplementary Figure 4. Manhattan plot of iUC association (black) within Shelties across chr13 overlayed with 1Mb windows of gene expression (red). The pvalues for association and excess dysregulation are plotted on the y-axis in -log scale.

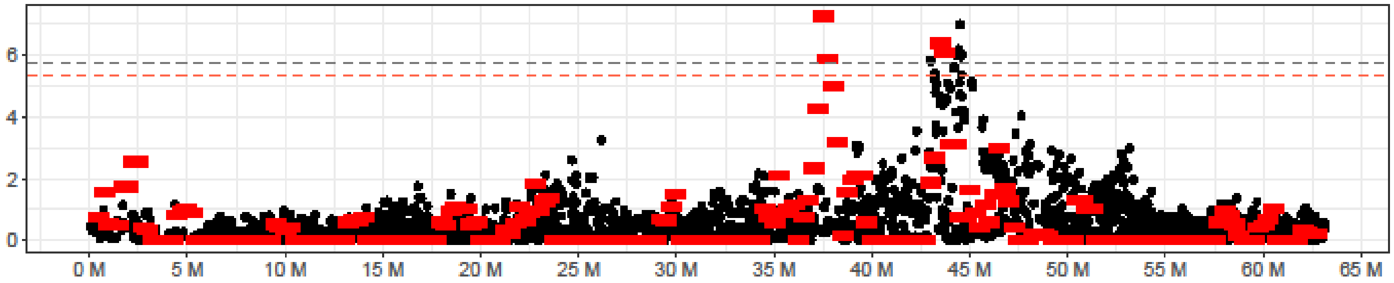

Supplementary Figure 5. Multiple alignment of the NIPAL1 protein sequence in eight diverse species. Conservation of the sequence is displayed using color with red indicating highly conserved, blue is less conserved, and grey is not conserved. Alignment graphed using the NCBI Blast multiple alignment tool, courtesy of the National Library of Medicine. The region of the gene which comprises the functional transport domain, amino acids 67 through 375, is shown above the alignment.

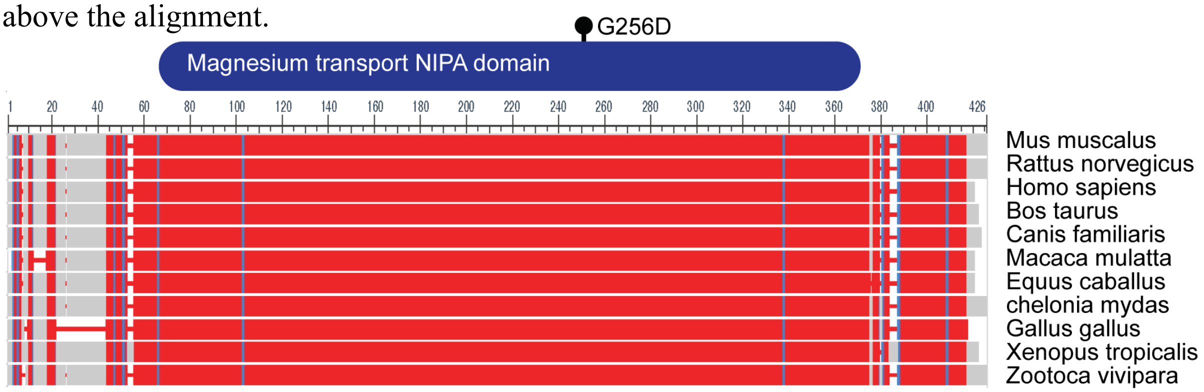

Supplementary Figure 6. Mixed breed analysis of atypical breed samples. Breed components are identified by excessive haplotype sharing with known pure-breeds. Haplotype sharing is calculated by pair with representatives of each breed and plotted with highest numbers toward the center of the graph. Dark grey shading indicates background sharing in which includes more than 95% of all across breed sharing. A. Atypical Border collie 1. B. Atypical Border collie 2

A. Atypical Border collie 1

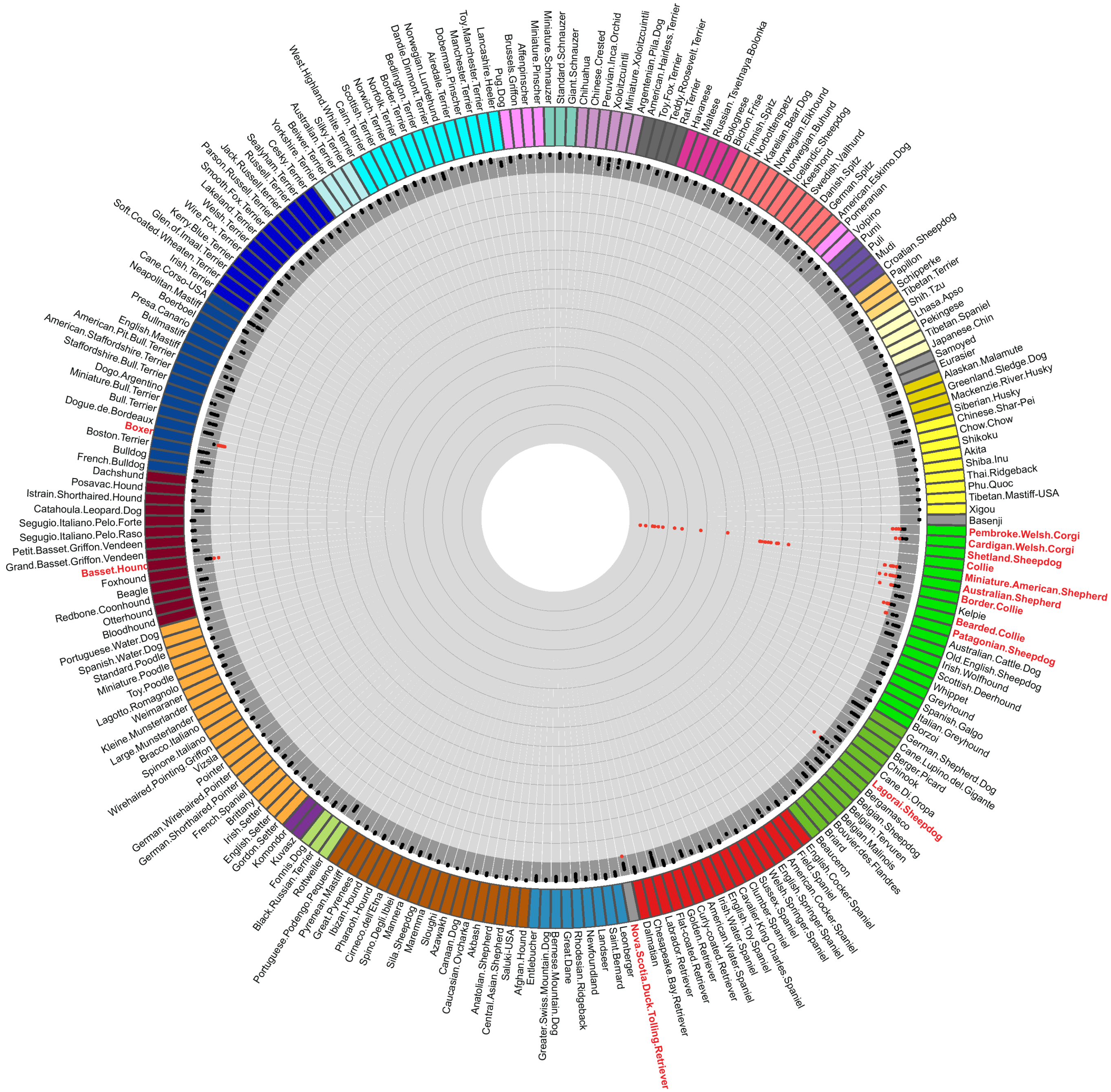

B. Atypical Border collie 2

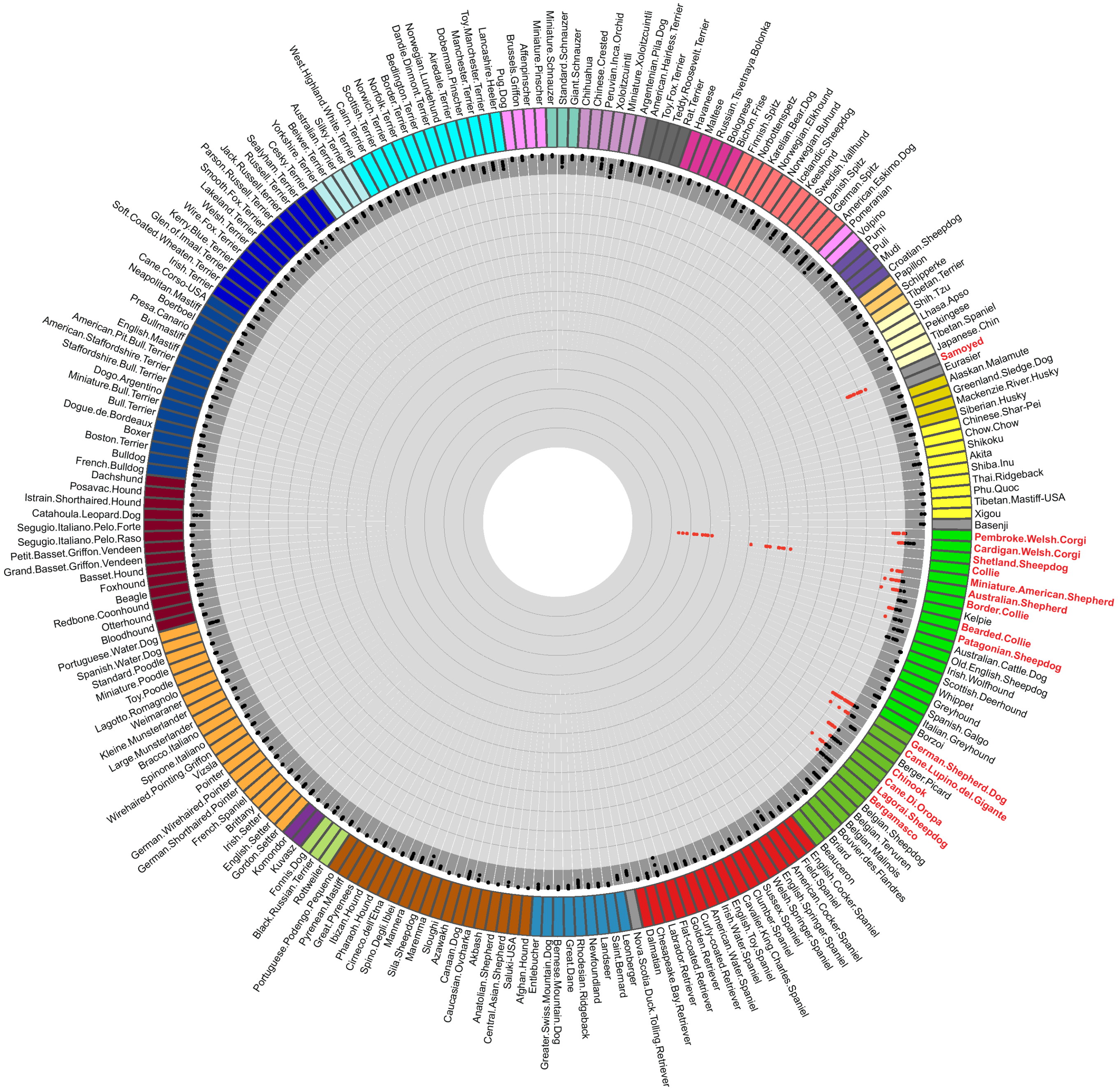

Supplementary Figure 7. Power by minor allele frequency. N=100, Case Ratio=.5, Alpha=0.05, OR=3 on the first row and OR=5 on the second row.

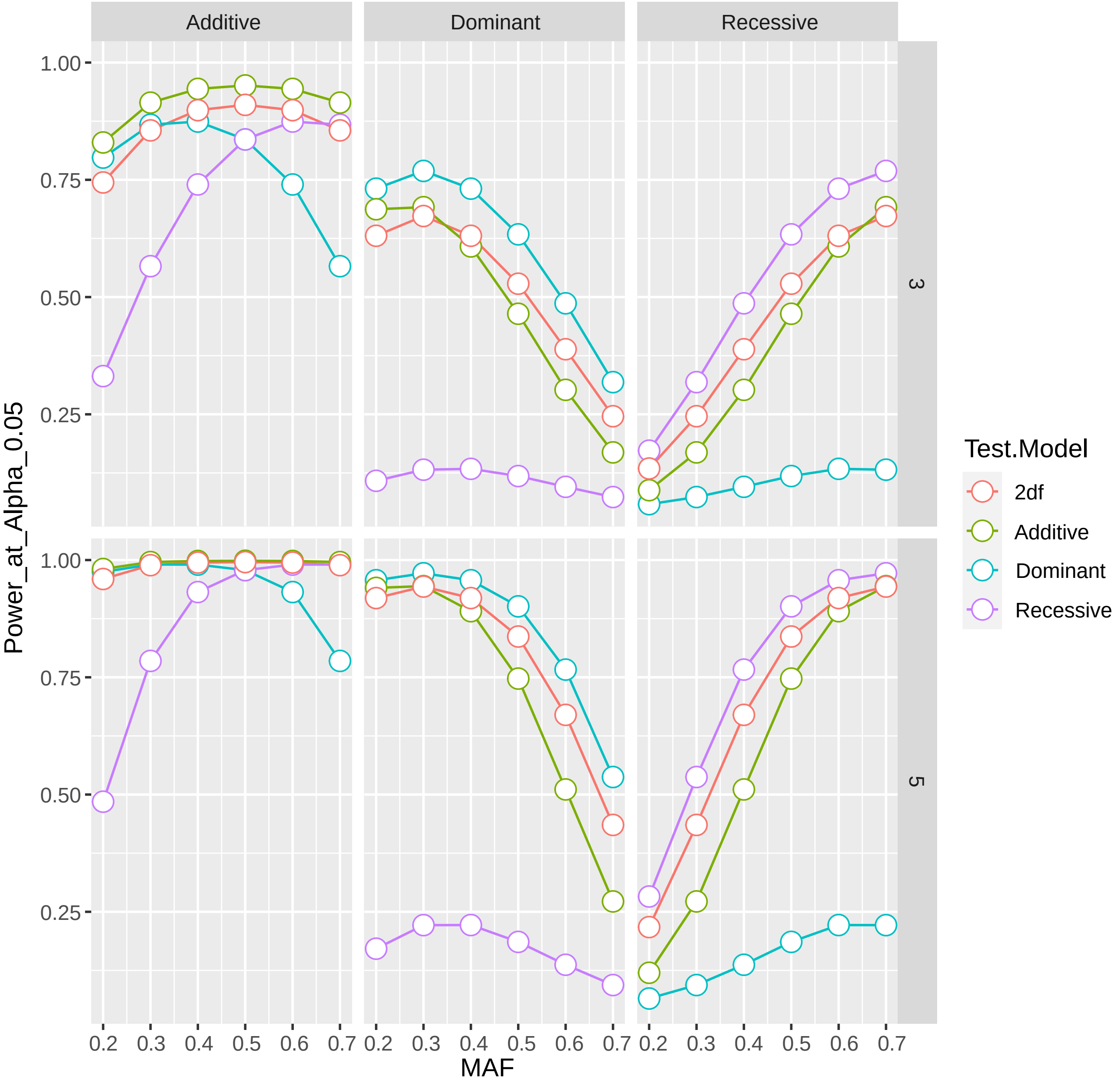

Supplementary Table 1. Metadata for 12 Shetland Sheepdog whole genome sequences.

NAME ID	Phenotype	Breed Name	BioProject	BioSample	BioExperiment	Platform	Sex	Age at collection
ShetlandSheepdog01	affected	Shetland Sheepdog	PRJNA288568	SAMN03801689	SRR2095504	Illumina HiSeq 2000	female	5.2
SSHP001	affected	Shetland Sheepdog	PRJNA685036	SAMN17192110	SRR13339206	Illumina NovaSeq 6000	male	n.d.
SSHP002	unaffected	Shetland Sheepdog	PRJNA685036	SAMN17192111	SRR13339205	Illumina NovaSeq 6000	female	13.1
SSHP003	unaffected	Shetland Sheepdog	PRJNA685036	SAMN17192112	SRR13339204	Illumina NovaSeq 6000	female	13
SSHP004	unaffected	Shetland Sheepdog	PRJNA685036	SAMN17192113	SRR13339203	Illumina NovaSeq 6000	male	15.3
SSHP005	unaffected	Shetland Sheepdog	PRJNA685036	SAMN17192114	SRR13339202	Illumina NovaSeq 6000	female	11.1
SSHP006	unaffected	Shetland Sheepdog	PRJNA685036	SAMN17192115	SRR13339201	Illumina NovaSeq 6000	male	11.4
SSHP007	affected	Shetland Sheepdog	PRJNA685036	SAMN17192116	SRR13339200	Illumina NovaSeq 6000	female	9
SSHP008	affected	Shetland Sheepdog	PRJNA685036	SAMN17192117	SRR13339199	Illumina NovaSeq 6000	female	7.8
SSHP009	affected	Shetland Sheepdog	PRJNA685036	SAMN17192118	SRR13339198	Illumina NovaSeq 6000	not reported	10.8
SSHP010	affected	Shetland Sheepdog	PRJNA685036	SAMN17192119	SRR13339197	Illumina NovaSeq 6000	female	8.5
SSHP011	affected	Shetland Sheepdog	PRJNA685036	SAMN17192120	SRR13339195	Illumina NovaSeq 6000	male	9.4

Supplementary Table 2. Associated SNPs spanning the chr13 locus including putative functional relevance.

position	effect	gene	enhancer targets	gene	lnc RNA	antisenseRNA	other RNA	human enhancer	H3K4me1	H3K27ac	H3K4me3	Associated Allele freq Dogs	Associated Allele freq Wild Canids
43469004	missense	ATP10D		✔								0.76	0.593
43469059	synonymous	ATP10D		✔								0.641	0.806
43469149	synonymous	ATP10D		✔								0.761	0.73
43473801	synonymous	ATP10D		✔								0.524	0.129
43480755	synonymous	ATP10D		✔								0.536	0.353
43499347	3'UTR	ATP10D		✔								0.735	0.814
43499759	3'UTR	ATP10D		✔								0.759	0.845
43499818	3'UTR	ATP10D		✔								0.709	0.757
43499936	3'UTR	ATP10D		✔								0.272	0.332
43500175	3'UTR	ATP10D		✔								0.735	0.614
43500193	3'UTR	ATP10D		✔								0.27	0.311
43500729	3'UTR	ATP10D		✔								0.734	0.604
43500852	3'UTR	ATP10D		✔								0.274	0.286
43501717	3'UTR	CORIN		✔								0.298	0.301
43501748	3'UTR	CORIN		✔								0.744	0.48
43501809	3'UTR	CORIN		✔								0.297	0.267
43502311	3'UTR	CORIN		✔								0.67	0.66
43552704	missense	CORIN		✔								0.43	0.646
43552764	synonymous	CORIN		✔								0.065	0.019
43556541	missense	CORIN		✔								0.07	0.092
43556551	synonymous	CORIN		✔								0.07	0.092
43556707	synonymous	CORIN		✔								0.611	0.832
43643605	missense	CORIN		✔								0.845	0.66
43643666	synonymous	CORIN		✔								0.813	0.597
43737556	5'UTR	CORIN		✔								0.246	0.456
43737605	5'UTR	CORIN		✔								0.248	0.456
43737733	5'UTR	CORIN		✔								0.808	0.981
43740727		CFRNASEQ_IGNC_00029792					✔					0.322	0.422
43740780		CFRNASEQ_IGNC_00029792					✔					0.84	0.873
43741262		CFRNASEQ_IGNC_00029792					✔	✔				0.336	0.238
43741534		CFRNASEQ_IGNC_00029792					✔					0.321	0.325
43742149		CFRNASEQ_IGNC_00029792					✔	✔				0.269	0.238
43742320		CFRNASEQ_IGNC_00029792					✔	✔				0.267	0.262
43742435		CFRNASEQ_IGNC_00029792					✔					0.268	0.233
43742584		CFRNASEQ_IGNC_00029792					✔					0.68	0.757
43742657		CFRNASEQ_IGNC_00029792					✔					0.776	0.874
43743061		CFRNASEQ_IGNC_00029792					✔					0.25	0.209
43743749	3'UTR	NFXL1		✔								0.771	0.813
43743799	3'UTR	NFXL1		✔		✔						0.769	0.97
43744490	3'UTR	NFXL1		✔		✔						0.769	0.796
43746022		CFRNASEQ_AS_00029793, RLOC_00021461				✔						0.757	0.312
43746351		CFRNASEQ_AS_00029793, RLOC_00021461				✔						0.253	0.162
43746876		CFRNASEQ_AS_00029793, RLOC_00021461				✔						0.254	0.157
43747059		CFRNASEQ_AS_00029793, RLOC_00021461				✔						0.771	0.804
43747142		CFRNASEQ_AS_00029793, RLOC_00021461				✔						0.757	0.308
43814824	5'UTR	NFXL1		✔				✔				0.01	0
43831167	3'UTR	CNGA1		✔								0.925	0.947
43854733											✔	0.609	0.107
43870715											✔	0.844	0.211

43870735											✔	0.792	0
43870965											✔	0.846	0.221
43876346			NIPAL1, CNGA1,NFXL1					✔	✔	✔		0.99	1
43878935											✔	0.356	0.783
43879074											✔	0.326	0.544
43879706											✔	0.371	0.912
43880281											✔	0.207	0.56
43880356											✔	0.224	0.99
43880374											✔	0.362	0.99
43880402											✔	0.367	0.99
43880453											✔	0.367	0.981
43880744											✔	0.184	0.186
43881552											✔	0.013	0
43882578											✔	0.987	1
43883655											✔	0.184	0.157
43897196	missense	NIPAL1		✔								0	0
44170736			SLAIN2,TEC,SLC10A4					✔	✔	✔		0.544	0.403
44170972			NIPAL1,NFXL1,SLAIN2,COMMD8,TEC,ZAR1,TXK,OCIAD2					✔	✔	✔		0.049	0
44171003			NIPAL1,NFXL1,SLAIN2,COMMD8,TEC,ZAR1,TXK,OCIAD2,CORIN,CWH43					✔	✔	✔		0.537	0.412
44171069			NIPAL1,NFXL1,SLAIN2,COMMD8,TEC,ZAR1,TXK,OCIAD2,CORIN,CWH43					✔	✔	✔		0.534	0.51
44171114			NIPAL1,NFXL1,SLAIN2,COMMD8,TEC,ZAR1,TXK,OCIAD2,CORIN,CWH43					✔	✔	✔		0.981	0.98
44171414			SLAIN2,SLC10A4,TEC,ZAR1,CORIN,NFXL1,NIPAL1,OCIAD2,TXK,					✔	✔	✔		0.652	0.465
44173539			CORIN,NIPAL1,TEC,SLAIN2,SLC10A4,ZAR1,OCIAD1,GABRA2,COMMD8,ATP10D					✔	✔	✔		0.894	0.985
44174029			SLAIN2,TEC,NIPAL1					✔	✔	✔		0.446	0.311
44174158			SLAIN2					✔	✔	✔		0.44	0.319
44174196			SLAIN2					✔	✔	✔		0.625	0.966
44174649			SLAIN2					✔	✔	✔		0.263	0.146
44174712			SLAIN2					✔	✔	✔		0.441	0.32
44174874			SLAIN2					✔	✔	✔		0.443	0.301
44200789			canine only						✔	✔	✔	0.598	0.976
44201406			canine only						✔	✔	✔	0.537	0.833
44201553			canine only						✔	✔	✔	0.442	0.314
44208747	synonymous	SLAIN2		✔								0.443	0.305
44246553	3'UTR	SLAIN2		✔								0.601	0.99
44246894	3'UTR	SLAIN2		✔								0.309	0
44246991	3'UTR	SLAIN2		✔								0.302	0.272
44247002	3'UTR	SLAIN2		✔								0.303	0.272
44261521		CFRNASEQ_IGNC_Single_00029807					✔					0.687	0.733

44263951		CFRNASEQ_IGNC_Single_00031791					✔					0.526	0.578
44264379		CFRNASEQ_IGNC_Single_00031791					✔					0.6	0.471
44264415		CFRNASEQ_IGNC_Single_00031791					✔					0.597	0.485
44264516		CFRNASEQ_IGNC_Single_00031791					✔					0.774	0.456
44264776		CFRNASEQ_IGNC_Single_00031791					✔					0.5	0.392
44269847		CFRNASEQ_IGNC_Single_00029808					✔					0.524	0.432
44270421		CFRNASEQ_IGNC_Single_00029808					✔					0.514	0.379
44270425		CFRNASEQ_IGNC_Single_00029808					✔					0.514	0.379
44275101		CFRNASEQ_IGNC_Spliced_00029809			✔		✔					0.308	0
44281916		CFRNASEQ_IGNC_Spliced_00029810					✔					0.269	0.699
44282075		CFRNASEQ_IGNC_Spliced_00029810					✔					0.546	0.99
44282105		CFRNASEQ_IGNC_Spliced_00029810					✔					0.074	0.117
44289006		CFRNASEQ_IGNC_Spliced_00029809			✔							0.706	1
44289560		CFRNASEQ_IGNC_Spliced_00029809			✔							0.366	0.019
44298597	synonymous	SLC10A4		✔								0.777	0.85
44309123	3'UTR	FRYL		✔								0.998	1
44368712	synonymous	FRYL		✔					✔		✔	0.141	0.121
44566201		CFRNASEQ_IGNC_00029813					✔	✔	✔		✔	0.874	0.995
44568351		CFRNASEQ_IGNC_00029813					✔					0.291	0.466
44568527		CFRNASEQ_IGNC_00029813					✔					0.627	0.85
44568528		CFRNASEQ_IGNC_00029813					✔					0.627	0.85
44568623		CFRNASEQ_IGNC_00029813					✔					0.628	0.853
44586896			NFXL1,TXK,FRYL,OCIAD1,OCIAD2					✔	✔	✔		0.837	0.971
44587111			NFXL1,TXK,FRYL,OCIAD1,OCIAD2					✔	✔	✔		0.546	0.422
44587126			NFXL1,TXK,FRYL,OCIAD1,OCIAD2					✔	✔	✔		0.546	0.427
44607491			FRYL, OCIAD1,OCIAD2					✔	✔	✔		0.349	0.233
44607797			FRYL, OCIAD1,OCIAD2					✔	✔	✔		0.767	0.886
44639641	3'UTR	OCIAD2		✔								0.213	0.068
44640770	3'UTR	OCIAD2		✔								0.949	0.966
44647520	synonymous	OCIAD2		✔				✔				0.277	0.291
44663006	missense	OCIAD2		✔								0.591	0.558
44663109	missense	OCIAD2		✔								0.594	0.345
44663157	missense	OCIAD2		✔								0.594	0.345
44713407		CFRNASEQ_IGNC_Spliced_00029822_1			✔							0.108	0.316
44714017		CFRNASEQ_IGNC_Spliced_00029822_1			✔							0.111	0.442
44714052		CFRNASEQ_IGNC_Spliced_00029822_1			✔							0.103	0.117
44714098		CFRNASEQ_IGNC_Spliced_00029822_1			✔							0.102	0.107

44714135		CFRNASEQ_IGNC_Spliced_00029822_1			✔							0.101	0.039
44714256		CFRNASEQ_IGNC_Spliced_00029822_1			✔							0.101	0.136

Supplementary Table 3. Predicted transcription factor binding sites created by the G>A transition at chr13: 44170972 within a likely multi-gene enhancer element.

TFbind ID	JASPAR ID	TF	sense	site	avgTPM*	score
	MA1137.1	FOSL1::JUNB	+	CGATGAGACACGT	38	0.832969
	MA1137.1	FOSL1::JUNB	-	ACGTGTCTCATCG	38	0.838318
	MA1142.1	FOSL1::JUND	+	GATGAGACAC	38	0.862818
	MA1132.1	JUN::JUNB	+	GATGAGACAC	165	0.854736
	MA0099.3	FOS::JUN	-	GTGTCTCATC	165	0.887599
M00174		V$AP1_Q6	+	GATGAGACACG	211	0.802181
M00174		V$AP1_Q6	-	CGTGTCTCATC	211	0.783753
M00199		V$AP1_C	+	ATGAGACAC	211	0.821283
M00199		V$AP1_C	-	GTGTCTCAT	211	0.880175
	MA1142.1	FOSL1::JUND	-	CGTGTCTCAT	38	0.858544
M00251		V$XBP1_01	-	CCTTAGTCGTGTCTCAT	188	0.806838
M00236		V$ARNT_01	+	TGAGACACGTCTAAGG	45	0.766628
	MA1566.1	TBX3	-	GACGTGTCTC	23	0.913377
M00119		V$MAX_01	+	GAGACACGTCTAAG	133	0.744371
M00119		V$MAX_01	-	CTTAGACGTGTCTC	133	0.744371
M00123		V$MYCMAX_02	-	TTAGACGTGTCT	91	0.795377
	MA0608.1	Creb3l2	+	GACACGTCT	59	0.953104
	MA0649.1	HEY2	+	GACACGTCTA	5	0.905384
MA0259.1		ARNT::HIF1A	-	AGACGTGT	45	0.951317
M00217		V$USF_C	+	ACACGTCT	347	0.803086
M00217		V$USF_C	-	AGACGTGT	347	0.803086
	MA0608.1	Creb3l2	-	TAGACGTGT	59	0.931579

* avgTPM=average transcripts per million. For complexes, avgTPM is given for the gene with the lowest expression level. Average expression across all JUN/FOS family members is provided for AP1 binding sites (range 38-542).

Supplementary Table 4. Secondary associated regions and the genes encompassed by the same.

NIPAL1 genotype	chr	position	P	region	size	Protein Coding Genes
AG	1	83750779	$1.36x10^{-6}$	chr1:83750779-84390848	640,069	ENSCAFG00000001773
AG	4	87441098	$4.52x10^{-7}$	chr4:87440198-87540261	100,063	FBXL7
AG	9	22495330	$6.23x10^{-7}$	chr9:22381987-23201516	819,529	WIPF2, RAPGEFL1, CASC3, MSL1, NR1D1, THRA, MED24, CSF3, PSMD3, GSDMA, LRRC3C, ORMDL3, GSDMB, ZPBP2, IKZF3, GRB7, **ERBB2**, MIEN1, PGAP3, PNMT, ENSCAFG00000028772, STARD3, PPP1R1B, NEUROD2, CDK12, MED1, FBXL20, ENSCAFG00000016470, STAC2, RL19_CANFA, ARL5C
GG	21	33595359	$1.67x10^{-7}$	chr21:31405230-33951574	2,546,344	OR5P2, OR10A6, ENSCAFG00000006866, ENSCAFG00000025199, NLRP10, EIF3F, TUB, RIC3, LMO1, STK33, TRIM66, RPL27A, ST5, AKIP1, C11orf16, ASCL3, TMEM9B, NRIP3, SCUBE2, TMEM41B, ENSCAFG00000007241, IPO7, ENSCAFG00000007332, ZNF143, **WEE1**, SWAP70, SBF2, ENSCAFG00000007532, ADML, AMPD3, ENSCAFG00000008138, ENSCAFG00000008290, AMPD3, RNF141, LYVE1, MRVI1, CTR9
AA	21	34085269	$5.64x10^{-7}$	chr21:33806003-34295586	489,583	MRVI1, CTR9, EIF4G2, ZBED5_CANFA
AA	28	34513379	$1.23x10^{-9}$	chr28:34513379-34747318	233,939	ENSCAFG00000032418
Positions giving in CanFam3.1. Genes extracted from the Broad improved annotation v1 Protein Coding Genes on the UCSC genome browser. Unknown proteins omitted.						

Supplementary Table 5. Predicted pathogenicity of conserved coding mutations.

	13:43897196		9:22775561	9:22775767	21:32843843
**Pathogenicity**					
Gene	NIPAL1		ERRB2	ERRB2	WEE1
Type	germline		somatic	somatic	somatic
Amino Acid Change	Gly256Asp		Asp277Tyr	Asp251His	Pro174Arg
CADD	**26.9**		**25.1**	**29.6**	**26.7**
DANN	**0.998**		**0.939**	**0.953**	**0.997**
ClinPred	**0.998**		**0.766**	**0.967**	**0.996**
fathmmMKL	**0.993**		**0.927**	**0.992**	**0.953**
PhD-SNP	**0.997**		**0.846**	**0.988**	**0.988**
PROVEAN	**0.932**		0.418	**0.899**	**0.924**
VEST	**0.912**		**0.688**	**0.925**	0.32
MutPred2	**0.85**		**0.66**	**0.697**	0.428
PolyPhen2	**1**		**0.988**	**0.975**	**1**
**Cancer specific function**					
CScape	**0.768**		**0.802**	**0.969**	**0.841**
CHASMplus	0.017		0.243	0.438	0.079
(p-value)	(0.691)		**(0.027)**	**(7.96E-04)**	(0.255)
CHASMplus bladder cancer	0.06		0.452	0.36	0.039
(p-value)	(0.234)		**(4.91E-05)**	**(0.000303)**	(0.375)
COSMIC count	317		2966	2966	377
COSMIC count urinary tract only	4		299	299	26

Significant predictions are bolded. The variant effect predictors DANN, ClinPred, fathmmMKL, PhD-SNP, PROVEAN, VEST, MutPred2, and PolyPhen2 score each variant on a scale from 0-1 with scores >0.5 indicating likely damaging or pathogenic mutations. CADD scores are provided on a phred scale with scores >20 in the top 1% of deleterious variation and >30 in the top 0.1%.

CScape uses a 0-1 scoring system to estimate oncogenic potential in mutations with 1 being most likely. The significance of cancer driver scores by CHASMplus are adjusted based on the frequency of the mutation therefore p-values are included with the score. COSMIC counts are all mutations that have been reported within the gene in the COSMIC database.
