# Supplementary material for: Genome-wide analyses reveals an association between invasive urothelial carcinoma in the Shetland sheepdog and NIPAL1
Source: NPJ Precis Oncol. 2024 May 22;8:112. doi: 10.1038/s41698-024-00591-0 (PMC11111773; doi:10.1038/s41698-024-00591-0)
Supplement: Supplementary file 2 — Reporting summary [file 41698_2024_591_MOESM2_ESM.pdf]

nature portfolio

Corresponding author(s): Dr Elaine Ostrander

Last updated by author(s): Apr 3, 2024

Reporting Summary

Nature Portfolio wishes to improve the reproducibility of the work that we publish. This form provides structure for consistency and transparency in reporting. For further information on Nature Portfolio policies, see our Editorial Policies and the Editorial Policy Checklist.

Statistics

For all statistical analyses, confirm that the following items are present in the figure legend, table legend, main text, or Methods section.

n/a	Confirmed	
☐	☒	The exact sample size (*n*) for each experimental group/condition, given as a discrete number and unit of measurement
☐	☒	A statement on whether measurements were taken from distinct samples or whether the same sample was measured repeatedly
☐	☒	The statistical test(s) used AND whether they are one- or two-sided<br>*Only common tests should be described solely by name; describe more complex techniques in the Methods section.*
☐	☒	A description of all covariates tested
☐	☒	A description of any assumptions or corrections, such as tests of normality and adjustment for multiple comparisons
☐	☒	A full description of the statistical parameters including central tendency (e.g. means) or other basic estimates (e.g. regression coefficient) AND variation (e.g. standard deviation) or associated estimates of uncertainty (e.g. confidence intervals)
☒	☐	For null hypothesis testing, the test statistic (e.g. *F*, *t*, *r*) with confidence intervals, effect sizes, degrees of freedom and *P* value noted<br>*Give P values as exact values whenever suitable.*
☒	☐	For Bayesian analysis, information on the choice of priors and Markov chain Monte Carlo settings
☒	☐	For hierarchical and complex designs, identification of the appropriate level for tests and full reporting of outcomes
☒	☐	Estimates of effect sizes (e.g. Cohen's *d*, Pearson's *r*), indicating how they were calculated

Our web collection on statistics for biologists contains articles on many of the points above.

Software and code

Policy information about availability of computer code

Data collection	Genome Studio 2.0.4<br>BWA v.0.7.1<br>GATK 4.3.0.0
Data analysis	pLINK v1.9<br>eigensoft v8.0.0<br>BEAGLE v4.1<br>Phylip v3.698<br>GEMMA v0.98<br>R v4.1<br>genpwr v1.0.4<br>Phase v2.1<br>samtools v.1.10<br>SNPeff v.5.2<br>VEP v.105<br>TFBind<br>AIModules<br>MACS2 v2.2.1

SICER2 v1.0.2
openCRAVAT v2.4.2
MutPred v.2.0
PolyPhen v.2
Sequencher v.5.4.6

For manuscripts utilizing custom algorithms or software that are central to the research but not yet described in published literature, software must be made available to editors and reviewers. We strongly encourage code deposition in a community repository (e.g. GitHub). See the Nature Portfolio guidelines for submitting code & software for further information.

Data

Policy information about availability of data

All manuscripts must include a data availability statement. This statement should provide the following information, where applicable:

- Accession codes, unique identifiers, or web links for publicly available datasets
- A description of any restrictions on data availability
- For clinical datasets or third party data, please ensure that the statement adheres to our policy

The datasets presented in this study can be found in public online repositories. Specifically: Illumina SNP chip data and ChIPseq data from cell lines has been submitted to the NCBI GEO database under accession GSE241367 and GSE254079 respectively. Whole tumor sequence is in the NCBI short read archive (SRA) under BioProject PRJNA1007700. Whole genome sequence can be found in the SRA under BioProjects PRJNA288568 and PRJNA685036. RNAseq was previously submitted to the SRA under BioProjects PRJNA559406 and PRJNA308949.

Research involving human participants, their data, or biological material

Policy information about studies with human participants or human data. See also policy information about sex, gender (identity/presentation), and sexual orientation and race, ethnicity and racism.

Reporting on sex and gender	This information has not been collected. Only animals were used in this study.
Reporting on race, ethnicity, or other socially relevant groupings	This information has not been collected. Only animals were used in this study.
Population characteristics	This information has not been collected. Only animals were used in this study.
Recruitment	This information has not been collected. Only animals were used in this study.
Ethics oversight	This information has not been collected. Only animals were used in this study.

Note that full information on the approval of the study protocol must also be provided in the manuscript.

Field-specific reporting

Please select the one below that is the best fit for your research. If you are not sure, read the appropriate sections before making your selection.

☒ Life sciences ☐ Behavioural & social sciences ☐ Ecological, evolutionary & environmental sciences

For a reference copy of the document with all sections, see nature.com/documents/nr-reporting-summary-flat.pdf

Life sciences study design

All studies must disclose on these points even when the disclosure is negative.

Sample size	Sample size was determined based on previous studies showing that 100 animals is a sufficient sample size to find variants associated with traits within a single dog breed. We also performed power analysis to determine the likelihood of finding association with loci of moderate to strong effect using 100 samples. Power was sufficient to continue with the analysis with the knowledge that we may miss variants of low effect or low penetrance.
Data exclusions	Samples were excluded from the analysis if they were determined to not be member of the breed included in the study.
Replication	Sufficient samples were not available to replicate the association analysis therefore we used related breeds to confirm disease association at the primary locus and all-breed population data to check for the presence and frequency of the mutations identified.
Randomization	Samples were collected as they presented in the clinic, from volunteers at dog competitions or from owners responding to advertisements. All

Randomization	samples were included equally with filtering based only on the quality of the diagnosis and the relationships between individuals. We then corrected for population structure to remove any further confounders.
Blinding	All samples are given a unique number when entering a study and all identifying information is removed. Only the data necessary to perform the analysis is retained.

Reporting for specific materials, systems and methods

We require information from authors about some types of materials, experimental systems and methods used in many studies. Here, indicate whether each material, system or method listed is relevant to your study. If you are not sure if a list item applies to your research, read the appropriate section before selecting a response.

Materials & experimental systems

n/a	Involved in the study
☐	☒ Antibodies
☐	☒ Eukaryotic cell lines
☒	☐ Palaeontology and archaeology
☐	☒ Animals and other organisms
☒	☐ Clinical data
☒	☐ Dual use research of concern
☒	☐ Plants

Methods

n/a	Involved in the study
☐	☒ ChIP-seq
☒	☐ Flow cytometry
☒	☐ MRI-based neuroimaging

Antibodies

Antibodies used	H3K4me1 rabbit polyclonal-AbCam, #ab8895<br>H3K4me3 rabbit polyclonal-AbCam, #ab8580<br>H3K27ac rabbit polyclonal AbCam #ab4729<br>IgG rabbit, AbCam #46540
Validation	According to the manufacturer these antibodies are designed for ChIPseq and have been tested suitable for ChIPseq and other immunoprecipitation experiments. The are predicted to work with all mammals, and have been used successfully in dogs (see Evans et al. 2021, PLOS Genetics 17(5): e1009543)

Eukaryotic cell lines

Policy information about cell lines and Sex and Gender in Research

Cell line source(s)	Cell lines were provided by the Knapp lab from the Werling Comparative Oncology Research Center at Purdue University, College of Veterinary Medicine.
Authentication	Authentication methods and results are described in Dhawan et al. Urol Oncol. 2009 May-Jun;27(3):284-92
Mycoplasma contamination	Because we used shared bio-safety cabinets, all cell lines are treated for Mycoplasma contamination prophylactically using Plasmocin (ant-mpt, Invivogen)
Commonly misidentified lines (See ICLAC register)	none

Animals and other research organisms

Policy information about studies involving animals; ARRIVE guidelines recommended for reporting animal research, and Sex and Gender in Research

Laboratory animals	The study did involve laboratory animals
Wild animals	The study did not involve wild animals.
Reporting on sex	Cases and controls in the association study were matched for sex.
Field-collected samples	Field collected samples were obtained at dog competition events. These were day trips, no housing was required and samples were kept at room temperature then transferred to 4 degree C. cooler until extraction.
Ethics oversight	Sample collection was performed following Animal Care and Use protocols associated with the collecting institutions (NIH Protocol GFS-05-1 and Purdue Protocol 1111000169).

Note that full information on the approval of the study protocol must also be provided in the manuscript.

Plants

Seed stocks
NA

Novel plant genotypes
NA

Authentication
NA

ChIP-seq

Data deposition

☒ Confirm that both raw and final processed data have been deposited in a public database such as GEO.

☒ Confirm that you have deposited or provided access to graph files (e.g. BED files) for the called peaks.

Data access links
May remain private before publication.
Data has been submitted to GEO under accession number GSE254079.

Files in database submission
K9TCC_Ab_Input_1.fastq
K9TCC_Ab_Input_2.fastq
K9TCC_Ab_H3K4me1_1.fastq
K9TCC_Ab_H3K4me1_2.fastq
K9TCC_Ab_H3K4me3_1.fastq
K9TCC_Ab_H3K4me3_2.fastq
K9TCC_Ab_H3K27ac_1.fastq
K9TCC_Ab_H3K27ac_2.fastq
K9TCC_Mx_Input_1.fastq
K9TCC_Mx_Input_2.fastq
K9TCC_Mx_H3K4me1_1.fastq
K9TCC_Mx_H3K4me1_2.fastq
K9TCC_Mx_H3K4me3_1.fastq
K9TCC_Mx_H3K4me3_2.fastq
K9TCC_Mx_H3K27ac_1.fastq
K9TCC_Mx_H3K27ac_2.fastq
K9TCC_Ab_H3K4me1_peaks.narrowPeak
K9TCC_Ab_H3K4me3_peaks.narrowPeak
K9TCC_Ab_H3K27ac_peaks.narrowPeak
K9TCC_Mx_H3K4me1_peaks.narrowPeak
K9TCC_Mx_H3K4me3_peaks.narrowPeak
K9TCC_Mx_H3K27ac_peaks.narrowPeak
K9TCC_Ab_H3K4me1_islandfiltered-normalized.wig
K9TCC_Ab_H3K4me3_islandfiltered-normalized.wig
K9TCC_Ab_H3K27ac_islandfiltered-normalized.wig
K9TCC_Mx_H3K4me1_islandfiltered-normalized.wig
K9TCC_Mx_H3K4me3_islandfiltered-normalized.wig
K9TCC_Mx_H3K27ac_islandfiltered-noramlized.wig

Genome browser session
(e.g. UCSC)
https://genome.ucsc.edu/s/hgparker/Chip_iUC

Methodology

Replicates
No replicates were performed thus peaks locations were compared between two independent samples and two calling methods.

Sequencing depth
library, reads, mapped reads (unique), read size, paired or single
K9TCC-Mx_input, 55709098, 55338295, 101, paired
K9TCC-Mx_H3K4me3, 59734738, 58192728, 101, paired
K9TCC-Mx_H3K27ac, 52779686, 52179405, 101, paired
K9TCC-Mx_H3K4me1, 57695670, 56863983, 101, paired
K9TCC-Ab_H3K4me1, 52358842, 51648408, 101, paired
K9TCC-Ab_input, 54402606, 54052522, 101, paired
K9TCC-Ab_H3K27ac, 50909450, 50421283, 101, paired
K9TCC-Ab_H3K4me3, 51204598, 50038402, 101, paired

Antibodies	H3K4me1 rabbit polyclonal-AbCam, #ab8895<br>H3K4me3 rabbit polyclonal-AbCam, #ab8580<br>H3K27ac rabbit polyclonal AbCam #ab4729
Peak calling parameters	sicer -t sample.bed -c input.bed -s cf31 -w 200 -g 200 -rt 1 -egf 0.74 -fdr 0.01<br>macs2 -t sample.bam -c input.bam -f BAM -g 2.5e+9 -B --verbose 3 -n sample_markers --outdir /data/
Data quality	Only peaks with an FDR <=1% were retained in the analysis
Software	Reads were aligned to the CanFam3.1 reference sequence using BWA-MEM and sorted with samtools.<br>Peaks were called using SICER2 and Macs2. Bedtools was used to convert .bam files to .bed files.
